# Machine learning model integrating oral microbiota and clinical features for predicting osteoporosis and bone loss in high-altitude populations

**DOI:** 10.1186/s12866-026-04718-0

**Published:** 2026-03-05

**Authors:** Jian Wang, Fen Yang, Sifan Hao, Changchao Dong, Yunru Tian, Yihang Xu, Shujuan Yang, Hui Yang, Xiong Xiao, Tianli Zheng, Haojiang Zuo, Xiaofang Pei, Xing Zhao

**Affiliations:** 1 West China School of Public Health and West China Fourth Hospital, Sichuan University, Chengdu, Sichuan 610041 China; 2 Health Promotion and Food Nutrition & Safety Key Laboratory of Sichuan Province, Sichuan University, Chengdu, Sichuan 610041 China; 3 West China School of Stomatology, Sichuan University, Chengdu, Sichuan 610041 China; 4 West China-PUMC C.C. Chen Institute of Health, Sichuan University, Chengdu, Sichuan 610041 China; 5 Innovation Center of Nursing Research, Nursing Key Laboratory of Sichuan Province, West China Hospital, Sichuan University, Chengdu, Sichuan 610041 China

**Keywords:** Osteoporosis, Bone loss, Machine learning, Oral microbiota, Tooth brush frequency, Tibetan population, High-altitude

## Abstract

**Background:**

Osteoporosis and bone loss (OP&BL) are major public health challenges, especially in high-altitude environments with chronic hypoxia. Current diagnostic methods, based on low-altitude populations, are impractical for large-scale screening in resource-limited, high-altitude settings. This study developed a machine learning-based predictive model for OP&BL by integrating oral microbiota data with clinical and questionnaire variables.

**Methods:**

We analyzed data from 560 Tibetan adults residing at high altitudes. Bone health status (OP&BL vs. normal) was determined by dual-energy X-ray absorptiometry. Oral microbiota profiles were characterized via 16 S rRNA sequencing. After feature selection using elastic net regression, five machine learning models, namely Logistic Regression (LR), Naïve Bayes (NB), Random Forest (RF), Support Vector Machine (SVM), and Extreme Gradient Boosting (XGB), were trained (60%, 337/560) and validated (40%, 223/560).

**Results:**

Feature selection identified nine predictors: Age, Gender, BMI, oral microbial genera *Abiotrophia*, Frequency of spicy food consumption (H23), Tooth brushing frequency (J5), Frequency of sweet-drink consumption (J3b), Current marital status (Separated/Divorced, A5_3), and frequency of numbing food consumption (H27). The LR model demonstrated good and stable performance with an AUC of 0.885 (95% CI: 0.823–0.937) on the test set, along with good calibration and the highest net clinical benefit. SHAP analysis indicated that oral factors *Abiotrophia* and Tooth brushing frequency together accounted for nearly 10% of the model’s total predictive contribution.

**Conclusions:**

We developed a machine learning model integrating oral microbiota and clinical data for predicting OP&BL in people living above 3500 m. This model could offer a promising non-invasive tool for early screening in resource-limited settings and highlights the potential role of oral factors in high-altitude bone health.

**Supplementary Information:**

The online version contains supplementary material available at 10.1186/s12866-026-04718-0.

## Highlights

This study develops a machine learning model tailored for high-altitude populations, addressing the limitations of current diagnostic methods based on low-altitude data.

By integrating oral microbiota data with clinical features, we propose a novel, non-invasive prediction model for osteoporosis and bone loss, specifically designed for high-altitude environments.

We evaluated multiple machine learning algorithms, including Logistic Regression (LR), Naïve Bayes (NB), Random Forest (RF), Support Vector Machine (SVM), and Extreme Gradient Boosting (XGB).

The LR model demonstrated superior, generalizable performance with an AUC of 0.885 (95% CI: 0.823–0.937) on the test set, along with excellent calibration and clinical utility.

Key predictors included the oral microbiota genus *Abiotrophia* and Tooth brushing frequency, highlighting the potential combined relevance of oral hygiene and microbiome-host interactions for bone health.

**Supplementary Information:**

The online version contains supplementary material available at 10.1186/s12866-026-04718-0.

## Introduction

Osteoporosis is a systemic skeletal disorder characterized by reduced bone mass and deterioration of bone microarchitecture, leading to increased fragility and fracture risk [1, 2]. This condition poses a major public health threat, contributing significantly to disability, loss of independence, and mortality. Its prevalence rises sharply with age, affecting hundreds of millions worldwide [3]. Although osteoporosis is a global health concern, emerging evidence suggests that high-altitude populations may have unique risk profiles due to environmental factors, such as chronic hypoxia and nutritional challenges [4, 5], which are not adequately addressed by current diagnostic methods. Therefore, improving early identification and prevention strategies for osteoporosis and bone loss (OP&BL) is of critical importance.

The conventional diagnosis of osteoporosis primarily relies on bone mineral density (BMD) measurements using techniques such as dual-energy X-ray absorptiometry (DXA) and quantitative computed tomography (QCT). However, these methods and their diagnostic criteria have predominantly been developed and validated in sea-level populations, limiting their generalizability to high-altitude groups where environmental factors such as chronic hypoxia may alter bone metabolism and fracture risk [6–8]. Additionally, the limited healthcare resources and high equipment costs in remote high-altitude regions further hinder the application of these methods [9]. A recent systematic review also reported significant discrepancies between DXA and QCT in diagnosing osteoporosis, complicating the standardization of these techniques [10]. These limitations collectively underscore the urgent need for practical and accessible screening tools in these settings.

Growing evidence indicates that the high-altitude environment shapes distinctive oral microbiota compositions [11, 12]. Moreover, the non-invasive nature of saliva collection and the responsiveness of oral microbiota to environmental factors make it a promising biomarker source for population studies in high-altitude settings [12]. Given the complex relationships between high-altitude environments, oral microbiota, and bone health, advanced analytical approaches are needed. Machine learning has demonstrated excellent capability in integrating multidimensional data for disease prediction [13, 14], with recent applications in osteoporosis risk assessment achieving AUCs of 0.77–0.84 by combining clinical variables with biochemical markers [15, 16]. Therefore, integrating demographic, lifestyle, biochemical, and microbial data using machine learning approaches shows particular promise for high-altitude populations where traditional risk models may not adequately apply.

To address these research gaps, we analyzed data from Tibetan participants in the 2024–2025 follow-up survey of the China Multi-Ethnic Cohort (CMEC) study. Using machine learning methods, we developed a prediction model for OP&BL that incorporates demographic characteristics, clinical measurements, lifestyle factors, and oral microbiota data (16 S rRNA sequencing) specifically tailored for high-altitude populations. We further explored potential mediating relationships between lifestyle factors, oral microbiota, and bone health outcomes.

## Methods

### Study design and participants

The CMEC initially recruited 100,000 participants from five provinces in southwestern China using a multi-stage, stratified, cluster sampling approach to ensure the representativeness of its ethnic sub-cohorts [17–19]. This study utilized data from the follow-up survey conducted between 2024 and 2025, which included 20,000 participants selected according to the same inclusion and exclusion criteria as the baseline survey. All participants underwent face-to-face interviews, physical examinations, and laboratory testing.

For this analysis, we focused on Tibetan individuals aged ≥ 40 years at altitudes > 3,500 m who met the following criteria: (1) completed questionnaires, physical examinations, and laboratory biochemical tests; (2) provided valid saliva samples for 16 S rRNA sequencing. Exclusion criteria included: (1) inability to participate due to language barriers or unforeseen circumstances; (2) presence of electronic or metal implants that interfered with bioelectrical impedance analysis; (3) diagnoses of malignancies, mental disorders, severe trauma, or serious musculoskeletal disorders.

The study was approved by the Ethics Committee of Sichuan University (K2016038 and K2020022). Written informed consent was obtained from all participants.

### Assessment of OP&BL

OP&BL were diagnosed based on BMD measurements obtained using DXA. According to World Health Organization criteria, osteoporosis was defined as a T-score ≤ −2.5 SD, and bone loss was defined as a T-score between − 1.0 and − 2.5 SD at the lumbar spine or femoral neck. Participants with T-score > −1.0 were classified as having normal BMD.

### Sample collection, DNA extraction, and microbiota analysis

#### Oral sample collection

Saliva samples were collected by trained medical personnel [20, 21]. All participants fasted overnight prior to saliva collection. Approximately 3 mL of saliva was collected. Fresh samples were placed in a low-temperature transport box with ice and transported to the laboratory, and subsequently transferred to –80 °C for extended storage [22].

#### DNA extraction and 16 S rRNA gene sequencing

Total microbial genomic DNA was extracted using the FastPure Stool DNA Isolation Kit (MJYH, Shanghai, China) according to the manufacturer’s instructions. DNA quality and concentration were assessed by 1.0% agarose gel electrophoresis and a NanoDrop^®^ ND-2000 spectrophotometer (Thermo Scientific, USA). Purified DNA was stored at − 80 °C until further use.

The hypervariable V3–V4 region of the bacterial 16 S rRNA gene was amplified with the primer pair 338 F (5′-ACTCCTACGGGAGGCAGCAG-3′) and 806R (5′-GGACTACHVGGGTWTCTAAT-3′) [23–25] using a T100 Thermal Cycler (Bio-Rad, USA). Each 20 µL PCR reaction contained 4 µL of 5× FastPfu Buffer, 2 µL of 2.5 mM dNTPs, 0.8 µL of each primer (5 µM), 0.4 µL of FastPfu Polymerase, and 10 ng of template DNA, with ddH₂O added to volume. The thermal cycling protocol consisted of initial denaturation at 95 °C for 3 min; 27 cycles of denaturation at 95 °C for 30 s, annealing at 55 °C for 30 s, and extension at 72 °C for 45 s; followed by a final extension at 72 °C for 10 min and cooling at 4 °C. All samples were amplified in triplicate. PCR products were resolved on a 2% agarose gel, purified, and quantified using a Synergy HTX microplate reader (Biotek, USA) [21, 24, 26]. To prevent contamination, all personnel were trained and strictly followed the protocols to use sterile equipment and reagents to prevent cross-contamination, use high-quality DNA extraction kits, and negative controls alongside the samples throughout the process [27–29]. We also routinely decontaminated equipment and surfaces with appropriate cleaning solutions, and UV-treat rooms and equipment to destroy contaminant DNA [30]. Purified amplicons were pooled in equimolar ratios and subjected to paired-end sequencing (2 × 300 bp) on an Illumina NovaSeq 6000 platform [31] following standard protocols.

#### Amplicon sequence processing and bioinformatic analysis

After demultiplexing, raw sequences were quality-filtered using fastp (v0.19.6) [32] and merged with FLASH (v1.2.11) [33]. High-quality sequences were denoised into amplicon sequence variants (ASVs) using DADA2 [34] within the QIIME2 pipeline (v2020.2) [35]. To account for uneven sequencing depth, samples were rarefied to at least 20,000 sequences per sample, yielding an average Good’s coverage of 97.9%. Taxonomic assignment of ASVs was performed using a Naive Bayes classifier trained on the SILVA 16 S rRNA database (v138.2).

Alpha diversity metrics, including Chao1, ACE, Shannon, and Simpson, were used to assess and compare species richness and evenness. To estimate the similarities among groups, beta diversity was measured via principal coordinate analysis (PCoA). LEfSe analysis was used to visualize group-level differences between BL and NR groups. To validate and control for key covariates, MaAsLin2 was subsequently applied. Prior to modeling, the data were processed to address sparsity and compositionality: microbial features with a prevalence of less than 10% across all samples were excluded, and the filtered counts were Total Sum Scaled (TSS) normalized and log₂-transformed [36]. The model was used to identify associations between taxa abundance and bone loss while adjusting for Age, Gender, and BMI. Statistical analyses were conducted using SPSS software v23.0 (IBM Corp., Armonk, NY, United States) and R software version (4.0.3).

### Predictor variables and feature selection

Four categories of variables were initially considered: (a) physical examination measures (e.g., height, weight, blood pressure) [19]; (b) questionnaire-derived data (e.g., marital status, dietary habits such as frequency of spicy food consumption) as detailed in the Supplementary Materials [17]; (c) blood test results (e.g., white blood cell, hemoglobin, glycated hemoglobin) [37]; and (d) oral microbiota features (genera-level abundance) [20, 21, 26]. To select high-quality risk factors while avoiding multicollinearity between variables, the elastic-net regularized logistic regression (glmnet) was used to initially screen the factors influencing OP&BL [38]. We set the mixing parameter α = 0.5 and selected λ by 10-fold cross-validation (minimum CV error). To ensure model stability, categorical variables were dummy-coded, and all predictors, including continuous, binary, and dummy-coded variables, were examined to ensure their variance inflation factors remained below 3. To prevent data leakage, all feature selection procedures (elastic net regression) were performed exclusively on the training set (*n* = 337). The subsequent model training and hyperparameter tuning were also confined to this subset.

### Machine learning modeling and evaluation

Five machine learning algorithms including Logistic Regression (LR), Naive Bayes (NB), Random Forest (RF), Support Vector Machine (SVM), Extreme Gradient Boosting (XGB) were trained (60%) and evaluated (40%) to predict OP&BL. The cohort (*n* = 560, OP&BL = 123, NR = 437) was split into a stratified training set (60%) and a held-out test set (40%) while preserving outcome prevalence. The training set contained 337 (OP&BL = 74 (22.0%), NR = 263 (78.0%)) and the test set contained 223 (OP&BL = 49 (22.0%), NR = 174 (78.0%)). All model selection and tuning were performed on the training set. Model performance was assessed using the area under the receiver operating characteristic curve (AUC), accuracy, sensitivity, specificity, precision, and F1-score. Calibration curves and decision curve analysis (DCA) were used to evaluate clinical utility. To enhance model interpretability, SHapley Additive exPlanations (SHAP) analysis was applied to quantify and visualize the contribution of each predictor [39].

### Mediation analysis

Mediation analysis was performed using the R mediation package with 1000 bootstrap samples. Effects were reported as average causal mediation effect (ACME), average direct effect (ADE), total effect (TE), and the proportion mediated (Prop_Mediated). The final results identified significant mediating variables satisfying ACME_*P* < 0.05, providing data support for subsequent in-depth analysis and interpretation [40].

### Statistical analysis

Continuous variables were expressed as mean ± standard deviation and compared using t-tests or Wilcoxon rank-sum tests, as appropriate. Categorical variables were compared using chi-square or Fisher’s exact tests. A *P* value < 0.05 was considered statistically significant. Multiple testing was corrected using the false discovery rate (FDR) method. Statistical analysis was performed using RStudio (2024.12.0 + 467). Detailed methodological procedures and additional supporting results are provided in the Supplementary Information.

### Sensitivity analysis

To assess the robustness of the logistic regression model, a sensitivity analysis was performed by perturbing each continuous predictor by ± 2 standard deviations around its mean while keeping other variables fixed. Second, to evaluate model performance across disease stages, we conducted subgroup analyses comparing NR vs. BL and NR vs. OP. Then the resulting changes in predicted probabilities were averaged across the test set to evaluate the stability and directionality of each predictor’s effect [41]. Effects are reported as Δ probability per 1 SD increase. Standardized sensitivity curves for all predictors were inspected for monotonicity and non-crossing, confirming consistent directional effects.

## Results

### Baseline characteristics and oral microbiota overview

**Fig. 1:**
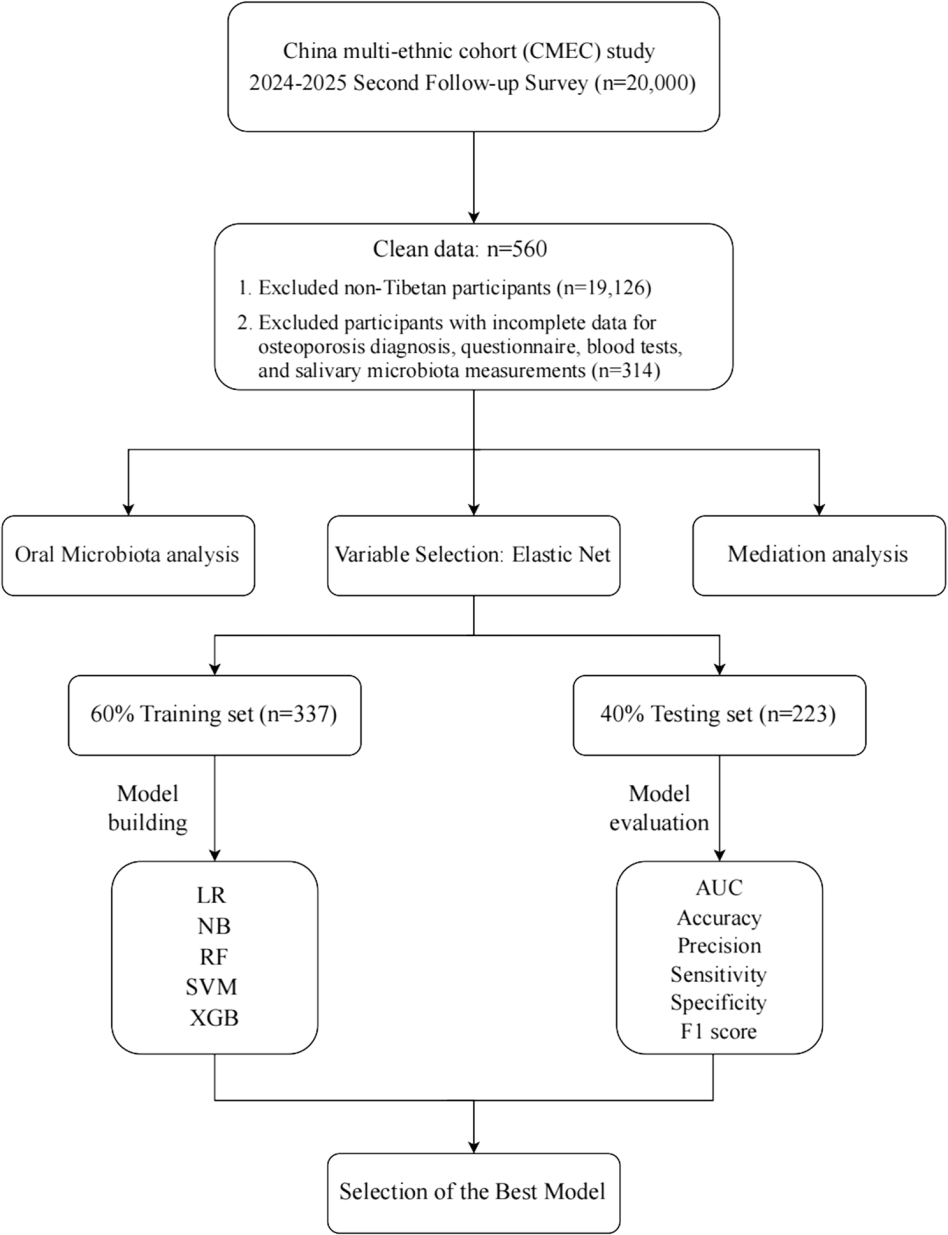
Flow chart of this study. LR, logistic regression; SVM, support vector machine; XGB, EXtreme Gradient Boosting; NB, naive Bayesian; RF, random forest; AUC, area under curve

From this cohort, we focused on Tibetan participants residing at altitudes above 3,500 m. After excluding non-Tibetan individuals and participants with incomplete osteoporosis diagnostic data, physical examinations, blood tests, or oral microbiota measurements, a total of 560 Tibetan participants were included. Participants were classified into three groups: osteoporosis (OP, *n* = 23), bone loss (BL, *n* = 100), and normal bone mineral density (NR, *n* = 437). Given the shared pathophysiology of bone loss and the limited sample size in the OP group, we combined the OP and BL groups into a single “OP&BL” group (*n* = 123) for subsequent machine learning analyses to enhance statistical power and focus on predicting any significant bone loss (Fig. 1).

Table 1 showed that participants in the OP&BL groups were significantly older than those in the NR group (49.8 ± 10.1 vs. 64.2 ± 8.6 years, *P* < 0.001) and females accounted for a higher proportion in the OP&BL groups (76.4% vs. 57.9%, *P* < 0.001). The Current marital status (Separated/Divorced, A5_3, *P* < 0.001) was also higher in the OP&BL group. In contrast, BMI (26.7 ± 4.13 vs. 24.9 ± 4.08, *P* < 0.001), Hemoglobin (Hb, 158 (148–175) vs. 152 (144–161), *P* < 0.001), Glycated Hemoglobin (HbA1C, 6.2(6.0–16.6) vs. 6.4(6.2–16.6), *P* < 0.001), and Frequency of eating spicy food in the past month (H23, *P* < 0.001), Self-assessed dental and oral condition (J1, *P* < 0.001), Frequency of sweet drinks consumption (J3b, *P* < 0.001), Tooth brushing frequency (J5, *P* < 0.001) were all significantly lower in the OP&BL groups compared with the NR group. No significant differences were observed in lymphocyte percentage (L), Neutrophil Percentage (N), Platelet Distribution Width (PDW), white blood cell count (WBC).

**Table 1:** Comparison of general characteristics of the group with normal and the group with osteoporosis and bone loss

Characteristics	Variables	NR (*N* = 437)	OP&BL (*N* = 123)	Overall (*N* = 560)	*P*_value
Demographic Characteistics	Gender				*P* < 0.001
	Male	184 (42.1%)	29 (23.6%)	213 (38.0%)	
	Female	253 (57.9%)	94 (76.4%)	347 (62.0%)	
	Age (years)^a^	49.8 ± 10.1	64.2 ± 8.58	53 ± 11.5	*P* < 0.001
	BMI(kg/m²) ^a^	26.7 ± 4.13	24.9 ± 4.08	26.3 ± 4.18	*P* < 0.001
	A5 (Current marital status)				*P* < 0.001
	1 (Married/Cohabiting)	382 (87.4%)	80 (65.0%)	462 (82.5%)	
	2 (Widowed)	12 (2.7%)	2 (1.6%)	14 (2.5%)	
	3 (Separated/Divorced)	24 (5.5%)	38 (30.9%)	62 (11.1%)	
	4 (Never Married)	19 (4.3%)	3 (2.4%)	22 (3.9%)	
	A9 (Including yourself, how many people live together in the household?)^b^	4 (3, 6)	4 (3, 5)	4 (3, 6)	*P* = 0.426
Routine blood test	EO (%)^b^	1.9 (1.1, 2.9)	2 (1.3, 3.4)	1.9 (1.2, 3.1)	*P* = 0.068
	Hb (g/L) ^b^	158 (148, 175)	152 (144, 161)	156 (146, 173)	*P* < 0.001
	HbA1C (%)^b^	6.2 (6.0, 6.6)	6.4 (6.2, 6.6)	6.3 (6, 6.6)	*P* < 0.001
	L (%)^a^	32.9 ± 7.86	33.3 ± 8.48	33.0 ± 8.00	*P* = 0.972
	N (%)^a^	58.5 ± 8.59	57.6 ± 9.19	58.3 ± 8.72	*P* = 0.719
	PLT (10^9/L) ^b^	245 (205, 284)	231 (195, 270)	242 (201, 283)	*P* = 0.044
	PDW (fL) ^b^	11.5 (10.2, 12.9)	11.2 (9.8, 12.9)	11.4 (10, 12.9)	*P* = 0.106
	WBC (10^9/L) ^b^	5.84 (5, 6.87)	5.91 (4.97, 6.71)	5.86 (5, 6.83)	*P* = 0.657
Dietary and Lifestyle Habits	H23 (Frequency of eating spicy food in the past month)				*P* < 0.001
	1 (Never/almost never)	125 (28.6%)	70 (56.9%)	195 (34.8%)	
	2 (A few times, but less than once a week)	49 (11.2%)	9 (7.3%)	58 (10.4%)	
	3 (Eat spicy food 1–2 days a week)	74 (16.9%)	14 (11.4%)	88 (15.7%)	
	4 (Eat spicy food 3–5 days a week)	41 (9.4%)	10 (8.1%)	51 (9.1%)	
	5 (Every day or almost every day)	148 (33.9%)	20 (16.3%)	168 (30.0%)	
	H27 (Frequency of eating numbing food in the past month)				*P* = 0.003
	1 (Never/almost never)	132 (30.2%)	59 (48.0%)	191 (34.1%)	
	2 (Ate a few times, but less than once a week)	68 (15.6%)	13 (10.6%)	81 (14.5%)	
	3 (Ate numbing food 1–2 days a week)	62 (14.2%)	12 (9.8%)	74 (13.2%)	
	4 (Ate numbing food 3–5 days a week)	45 (10.3%)	5 (4.1%)	50 (8.9%)	
	5 (Eat it every day or almost every day)	130 (29.7%)	34 (27.6%)	164 (29.3%)	
	H3 (One month’s consumption of salt/g)^b^	500 (250, 500)	400 (250, 500)	500 (250, 500)	*P* = 0.175
	J3b (Frequency of sweet drinks consumption)				*P* < 0.001
	1 (≥ 2 times per day)	3 (0.7%)	2 (1.6%)	5 (0.9%)	
	2 (1 time per day)	28 (6.4%)	3 (2.4%)	31 (5.5%)	
	3 (2–6 times per week)	81 (18.5%)	7 (5.7%)	88 (15.7%)	
	4 (1 time per week)	51 (11.7%)	13 (10.6%)	64 (11.4%)	
	5 (1–3 times per month)	82 (18.8%)	14 (11.4%)	96 (17.1%)	
	6 (Rarely/never)	192 (43.9%)	84 (68.3%)	276 (49.3%)	
Oral Health	J1 (Self-assessment of dental and oral condition)				*P* < 0.001
	1 (Very good)	48 (11.0%)	4 (3.3%)	52 (9.3%)	
	2 (Good)	125 (28.6%)	18 (14.6%)	143 (25.5%)	
	3 (Fair)	146 (33.4%)	44 (35.8%)	190 (33.9%)	
	4 (Poor)	98 (22.4%)	43 (35.0%)	141 (25.2%)	
	5 (Very bad)	20 (4.6%)	14 (11.4%)	34 (6.1%)	
	J5(Tooth brushing frequency)				*P* < 0.001
	1 (≥ 2 times per day)	86 (19.7%)	9 (7.3%)	95 (17.0%)	
	2 (1 time per day)	312 (71.4%)	82 (66.7%)	394 (70.4%)	
	3 (2–6 times per week)	19 (4.3%)	11 (8.9%)	30 (5.4%)	
	4 (1 time per week)	2 (0.5%)	2 (1.6%)	4 (0.7%)	
	5 (1–3 times per month)	4 (0.9%)	2 (1.6%)	6 (1.1%)	
	6 (Rarely/never)	14 (3.2%)	17 (13.8%)	31 (5.5%)	

a: Mean ± SD

b: Median (Q1, Q3); *NR* Normal, *OP&BL *osteoporosis and bone loss, *BMI *Body Mass Index (kg/m²), *EO *Eosinophil Percentage (%), *Hb *Hemoglobin (g/L), *HbA1C* Glycated Hemoglobin (%), *L *Lymphocyte Percentage (%), *N *Neutrophil Percentage (%), *PLT *Platelet Count (10⁹/L), *PDW *Platelet Distribution Width (fL), *WBC* White Blood Cell Count (10⁹/L); A5: Current marital status (1 = Married/Cohabiting, 2 = Widowed, 3 = Separated/Divorced, 4 = Never Married); A9: Household Size, number of people living together including oneself; H23: Frequency of eating spicy food in the past month (1 = Never/almost never, 2 = A few times but less than once a week, 3 = Eat spicy food 1–2 days a week, 4 = Eat spicy food 3–5 days a week, 5 = Every day or almost every day); H27: Frequency of eating numbing food in the past month (1 = Never/almost never, 2 = Ate a few times but less than once a week, 3 = Ate numbing food 1–2 days a week, 4 = Ate numbing food 3–5 days a week, 5 = Eat it every day or almost every day); H3: One-month consumption of salt (g); J1: Self-assessment of dental and oral condition (1 = Very good, 2 = Good, 3 = Fair, 4 = Poor, 5 = Very bad); J3b: Frequency of sweet drinks (1 = ≥ 2 times per day, 2 = 1 time per day, 3 = 2–6 times per week, 4 = 1 time per week, 5 = 1–3 times per month, 6 = Rarely/never); J5: Tooth brushing frequency (1 = ≥ 2 times per day, 2 = 1 time per day, 3 = 2–6 times per week, 4 = 1 time per week, 5 = 1–3 times per month, 6 = Rarely/never)

**Fig. 2:**
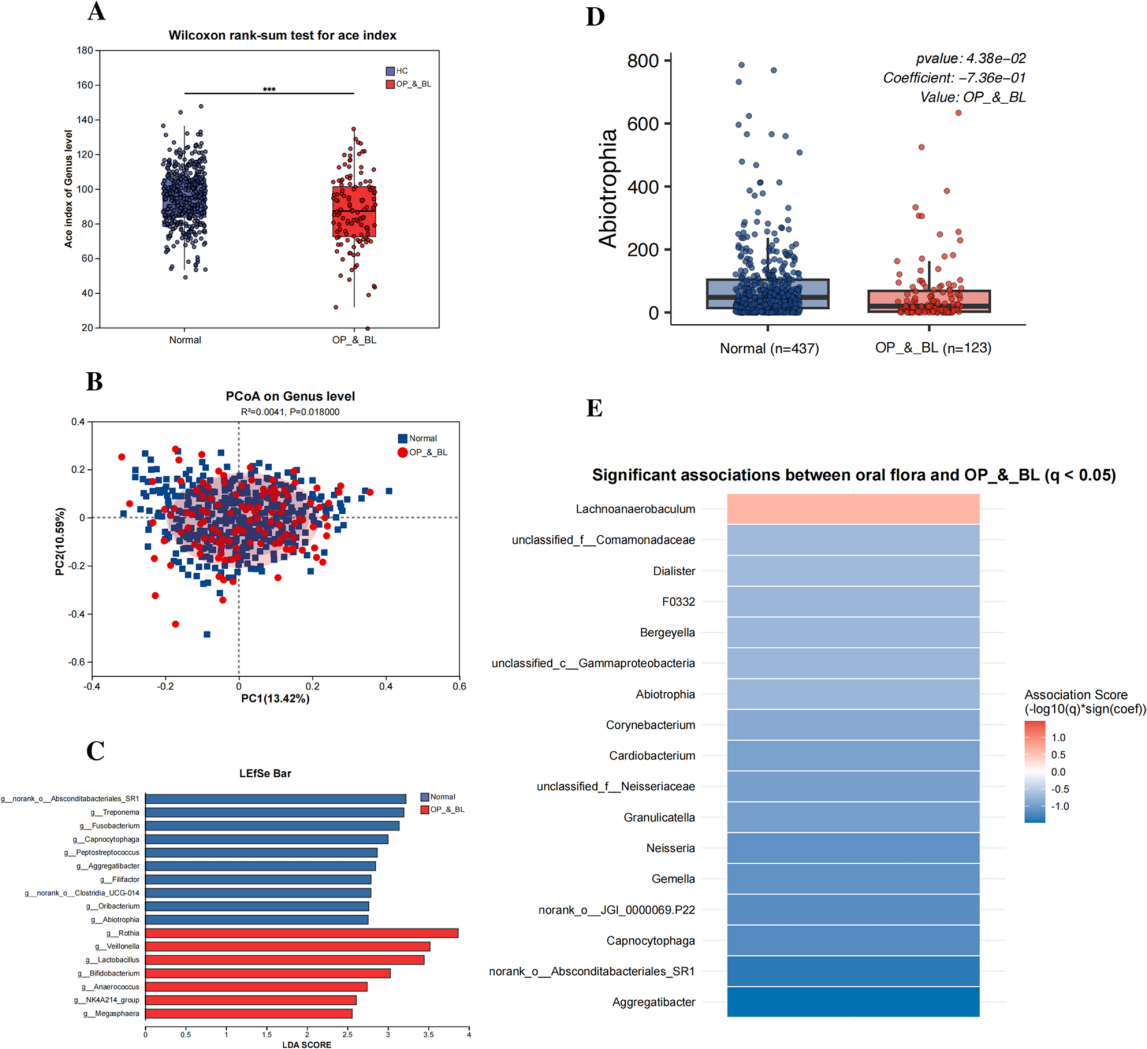
Alterations in oral microbiota diversity and composition associated with high-altitude OP&BL (*n*=560). **A** Alpha diversity analysis shows a significantly reduced ACE index in the osteoporosis and bone loss (OP&BL) and Normal (NR) groups (*P* < 0.001). **B** PCoA analysis also shows significant differences in microbial structure at the genus level (*P*=0.018). **C** LDA scores from LEfSe analysis show significant differences in OP&BL and NR groups at genus levels; highlights dominant bacteria including Rothia and Veillonella in the OP&BL group, Treponema and Abiotrophia in the NR group. **D** Boxplots from MaAsLin2 analysis show the relative abundance of the two genera significantly associated with OP&BL status after adjusting for Age, BMI, and Gender: Abiotrophia (coefficient = −0.736, *P* = 0.0438). **E** Heatmap visualizing the strength of association (log₁₀(*p*) × sign(coefficient)) for oral microbiota genera significantly associated with OP&BL status (MaAsLin2, adjusted for Age, BMI, and Gender; *q* <0.05 indicates False discovery rate (FDR)<0.05)

Analysis of oral microbiota following rarefaction to 23,033 sequences per sample to ensure even sequencing depth across all samples (Fig. S1, which shows the rarefaction curve confirming sequencing depth sufficiency) revealed significant differences in both alpha and beta diversity (*P* < 0.05, Fig. 2A and B). Furthermore, we identified specific microbial taxa associated with bone health status: LEfSe analysis revealed that *Rothia* and *Veillonella* were enriched in the OP&BL group, whereas *Treponema* and *Abiotrophia* were enriched in the NR group (Fig. 2C). To manage data sparsity and reduce false positives, we applied prevalence filtering (retaining genera present in ≥ 10% of samples; see prevalence diagnostics in Fig. S2). Using this filtered dataset, MaAsLin2 analysis, which controlled for key covariates (age, gender, BMI), also revealed significant associations for *Abiotrophia* with OP&BL status (Fig. 2D-E).

### Feature selection

**Fig. 3:**
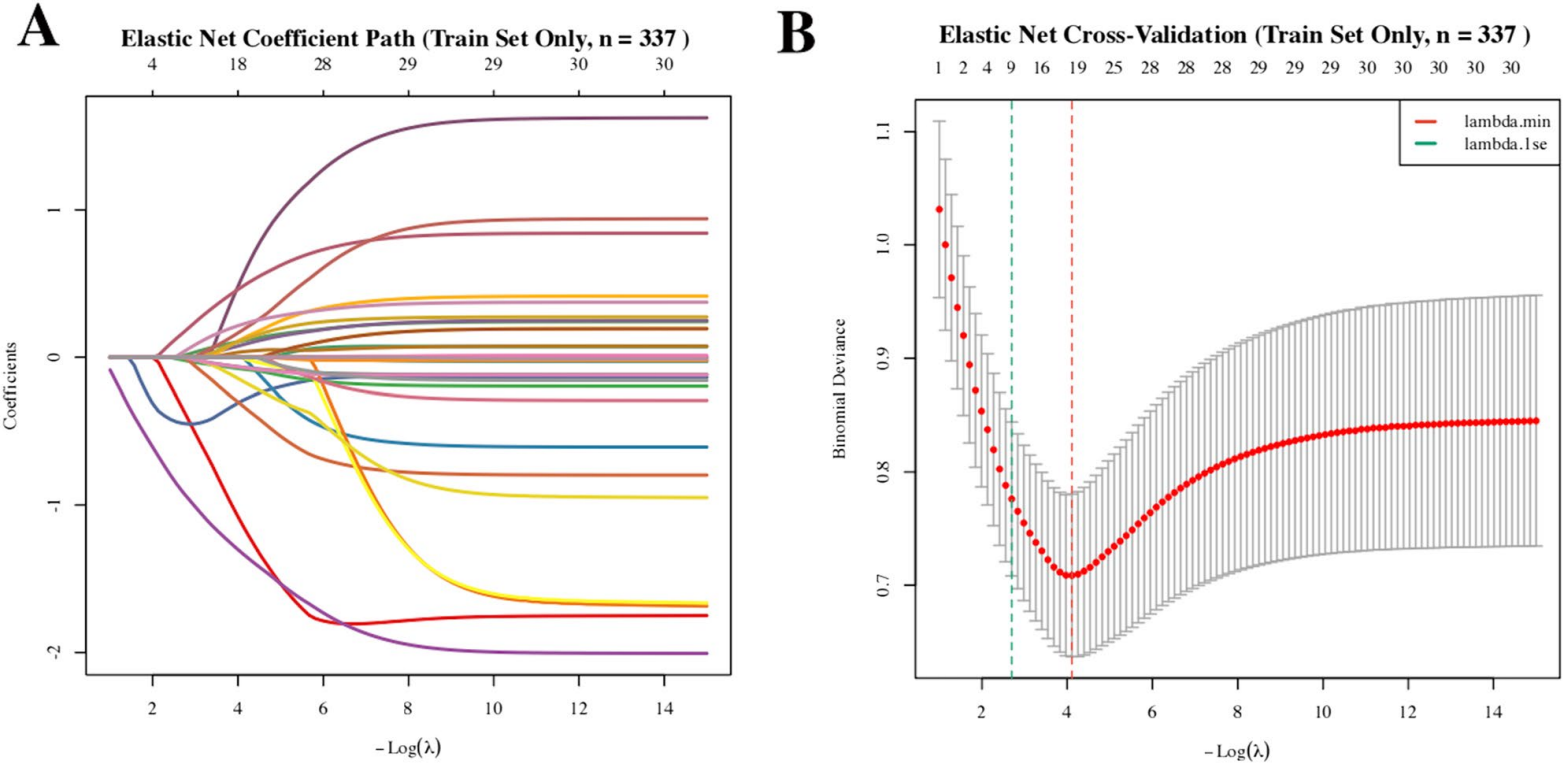
Elastic net regression for feature selection. **A** Elastic net coefficient path plot for 27 variables. **B** Cross-validation curve (10-fold cross validation). The left dashed line represents lambda.1se and the right dashed line represents lambda.min

To ensure the stability of subsequent modeling, we first assessed multicollinearity among all 27 candidate variables. The variance inflation factor (VIF) for each predictor remained below the threshold of 3 (Fig. S3), confirming the absence of severe multicollinearity. We performed feature selection using elastic net regression with 10-fold cross-validation. The coefficient path shows a monotonic decrease with log(λ), demonstrating a clear variable compression trajectory. Cross-validation selected λ.1se as the optimal penalty parameter, which retained 9 predictors from an initial set of 27 variables (Fig. 3A and B). Initially incorporating 27 candidate variables spanning physical examination (age, BMI), questionnaire data (A5, Current marital status), blood tests (Hb), and oral microbiota, where λ.1se was selected as the regularization parameter to balance model simplicity and stability, ultimately identifying nine robust predictors: Age, Gender, BMI, *Abiotrophia*, frequency of spicy food consumption (H23), Tooth brushing frequency (J5), Frequency of sweet-drink consumption (J3b), Current marital status (Separated/Divorced, A5_3), and Frequency of numbing food consumption (H27). The inclusion of the microbial taxa is consistent with the differential abundance results (Fig. 2), reinforcing their biological relevance to OP&BL.

Furthermore, the nine predictors retained after elastic net shrinkage showed only modest intercorrelations (max |r| = 0.37, Fig. S4, which presents the correlation matrix of the selected features), further confirming the absence of severe multicollinearity and supporting model validity.

### Model evaluation

**Fig. 4:**
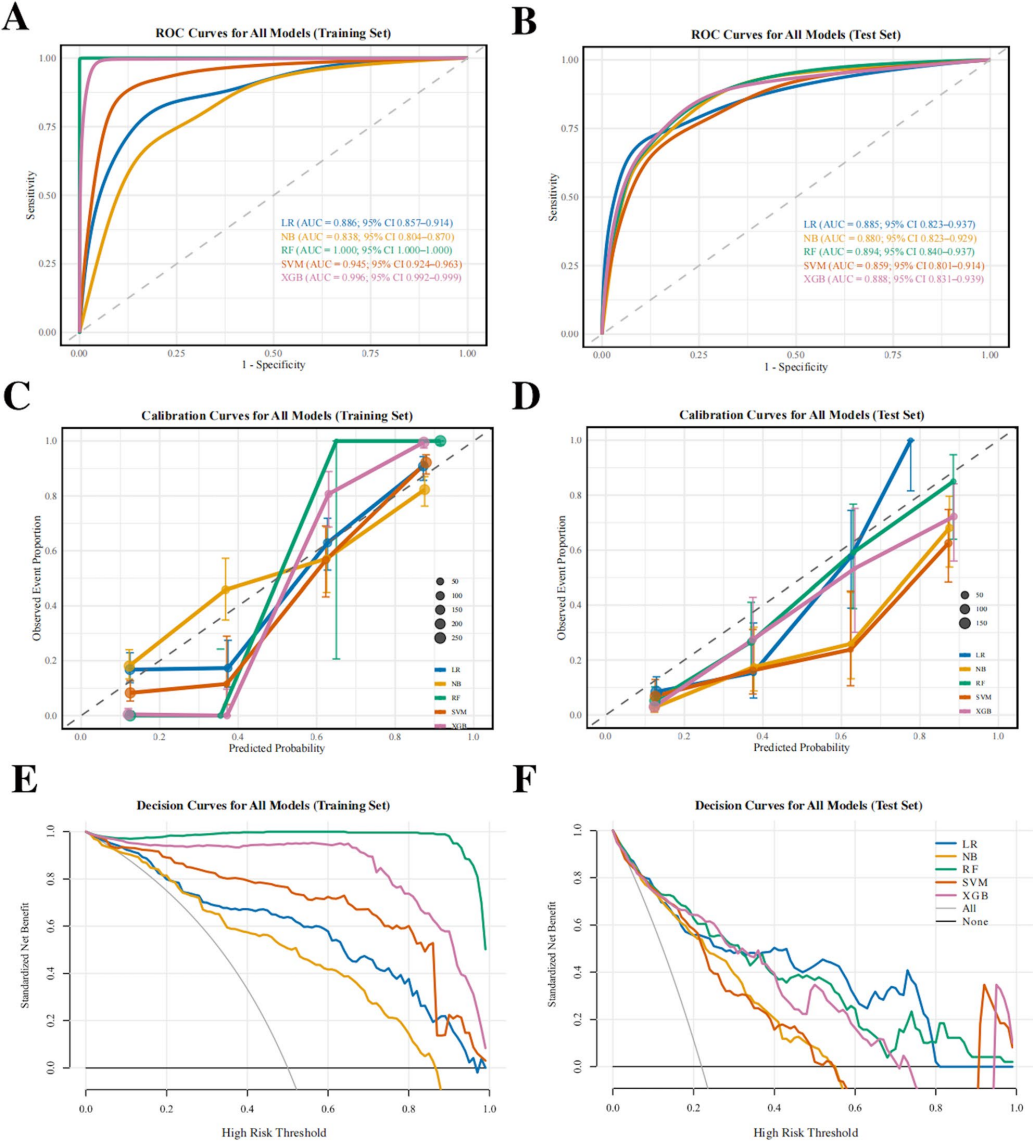
Machine learning models performance evaluation. ROC curves for the five models in the training set (**A**) and test set (**B**). Calibration curve for the five models in the training set (**C**) and test set (**D**). Decision curves for the five models in the training set (**E**) and test set (**F**). LR, logistic regression; NB, naive Bayesian; RF, random forest; SVM, support vector machine; XGB, EXtreme Gradient Boosting.

We constructed and evaluated five machine learning models to predict OP&BL status based on selected variables. On the training set (Fig. 4A), RF achieved a perfect AUC value of 1.000 (1.000–1.000), followed closely by XGB (AUC = 0.996, 0.992–0.999). SVM and LR also demonstrated high discriminative ability, with AUC values of 0.945 (0.924–0.963) and 0.886 (0.857–0.914), respectively, while NB had a moderate AUC of 0.838 (0.804–0.870). Notably, in the test set (Fig. 4B), LR maintained stable performance (AUC = 0.885, 0.823–0.937) and demonstrated excellent generalization, indicating an optimal balance between discrimination and robustness [42].

Calibration analysis (Fig. 4C and D) revealed that the LR model, after Platt scaling, was well-calibrated, with predicted probabilities closely aligning observed outcomes. This indicated that a combination of good discriminatory power and reliable risk estimation, underscoring its potential clinical utility.

Decision curve analysis (DCA) provided a clinical perspective on model utility. In the training set (Fig. 4E), across the entire clinically relevant risk threshold range (0–1), all five models produced positive net benefits and outperformed the “no treatment” and “all treatment” strategies. In the test set (Fig. 4F), LR maintained the highest and most stable net benefit at all thresholds, confirming its superior clinical applicability.

Table 2 shows the predictive power of five models in the training and test set, from which it can also be seen that LR has the strongest ability to discriminate between normal and OP&BL groups. The confusion matrix for all five models on the test set (Fig.S5) visually demonstrates that LR achieves the optimal balance between sensitivity (73.5%) and specificity (91.4%). Therefore, based on a comprehensive assessment of discrimination, calibration, and clinical benefit, LR was selected as the optimal model for predicting OP&BL risk.

**Table 2:** Comparison of the predictive power of several models in the train and test set

Model	Dataset	Accuracy	Sensitivity	Specificity	Precision	F1	AUC
LR	Train	0.819	0.837	0.802	0.809	0.822	0.886 (0.857–0.914)
LR	Test	0.874	0.735	0.914	0.706	0.720	0.885 (0.823–0.937)
NB	Train	0.753	0.738	0.768	0.761	0.749	0.838 (0.804–0.870)
NB	Test	0.798	0.796	0.799	0.527	0.634	0.880 (0.823–0.929)
RF	Train	1.000	1.000	1.000	1.000	1.000	1.000 (1.000–1.000)
RF	Test	0.861	0.612	0.931	0.714	0.659	0.894 (0.840–0.937)
SVM	Train	0.882	0.920	0.844	0.855	0.886	0.945 (0.924–0.963)
SVM	Test	0.785	0.714	0.805	0.507	0.593	0.859 (0.801–0.914)
XGB	Train	0.975	0.996	0.954	0.956	0.976	0.996 (0.992–0.999)
XGB	Test	0.857	0.694	0.902	0.667	0.680	0.888 (0.831–0.939)

*LR* logistic regression, *SVM *support vector machine, *XGB* extreme gradient boosting, *NB* naive Bayesian, *RF* random forest

Finally, 10-fold cross-validation confirmed the stability of LR model: mean AUC = 0.849 ± 0. 082 and Kappa = 0.443, with 90% of folds exceeding the clinically acceptable threshold of 0.75 and 70% surpassing 0.820 (Fig. S6, which illustrates the performance distribution across folds, supporting model robustness).

### Variable importance assessment

**Fig. 5:**
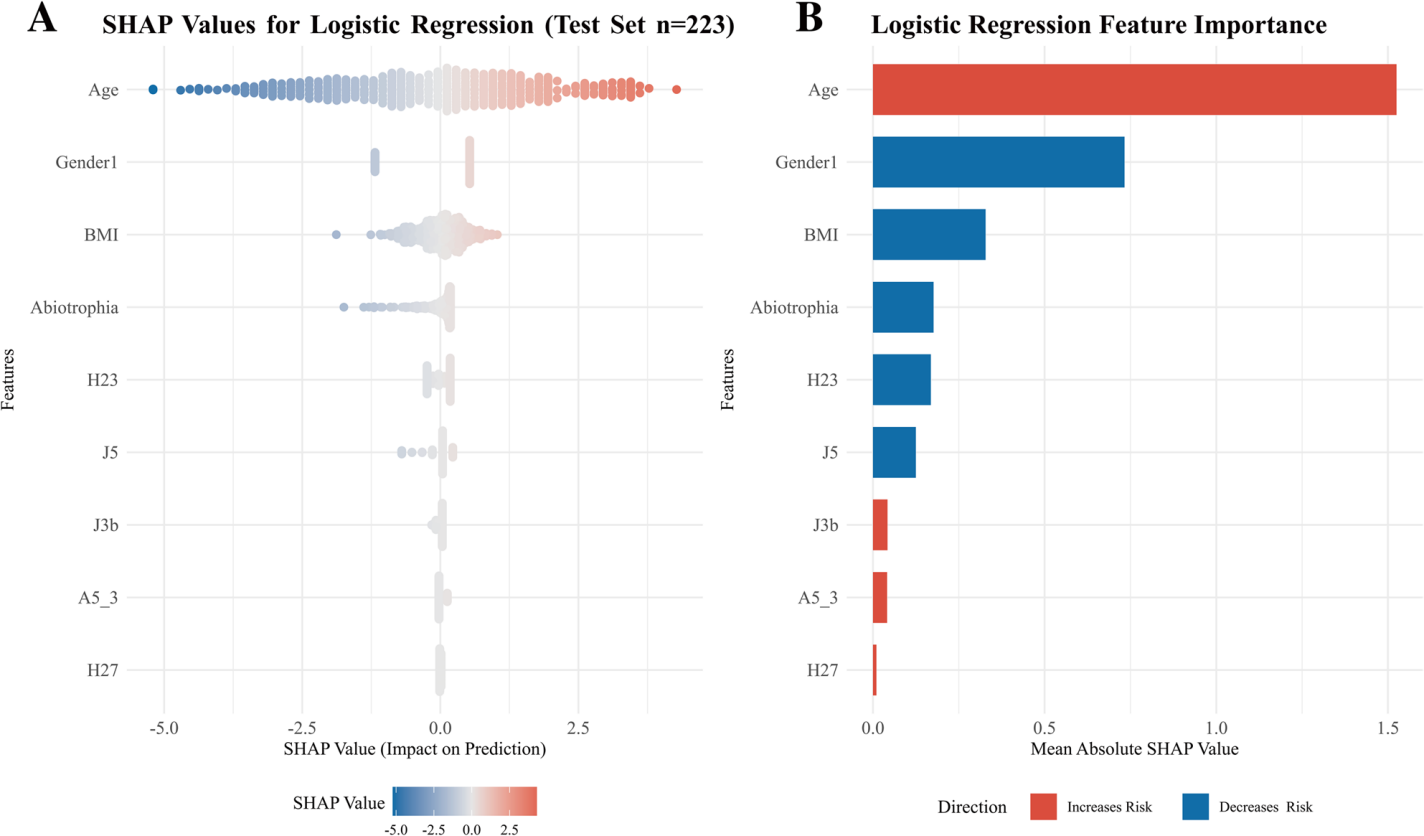
SHAP-based interpretation of the optimal Logistic Regression (LR) model. **A** Beeswarm plot showing the distribution of SHAP values per feature. Each point represents one participant. The position on the x-axis indicates the feature's impact on the predicted log-odds (positive values push towards OP&BL, negative towards normal), and color corresponds to the feature’s actual value (red: high, blue: low). This plot visualizes the direction, magnitude, and individualized effect of each predictor. **B** Feature importance ranking based on mean absolute SHAP values, illustrating the overall contribution (global importance) of each variable to the model’s predictions, independent of its original scale. Taken together, these visualizations demonstrate how SHAP analysis complements standard regression coefficients: it provides an intuitive ranking of global feature importance (**B**) alongside a detailed, personalized map of how each factor influences individual risk predictions (**A**)

SHAP analysis (Fig. 5A and B) was employed to interpret the contribution and directional influence of each predictor in the LR model. Age was identified as the most important predictor, with higher age associated with increased risk of OP&BL. Gender was the second most influential factor, with females exhibiting higher risk than males. BMI, *Abiotrophia*, Frequency of spicy food consumption (H23), and Tooth brushing frequency (J5) were associated with a protective effect, whereas frequent consumption of sweet drinks (J3b), Current marital status (Separated/Divorced, A5_3) and Frequency of numbing food consumption (H27) were associated with increased risk.

Consistent with these directional insights, the quantification of mean absolute SHAP (|SHAP|) values confirmed the dominance of Age (1.52), BMI (0.73), and Gender (0.33), which together accounted for over 65% of the model’s total predictive contribution. In comparison, the oral factors *Abiotrophia* (0.18) and tooth brushing frequency (0.12) together contributed nearly 10% of the total effect (Fig. 5B). The contributions of all other predictors were comparatively minor, each representing no more than 5% of the total.

### Analysis of mediating effects

Given the association of both spicy food consumption (H23) and oral microbiota with OP&BL risk in our model, we performed an exploratory mediation analysis to investigate their potential interplay. Before adjustment for confounders, exploratory mediation analysis suggested that *Abiotrophia* abundance might mediate 6.83% of the total effect (ACME = −0.00542, *P* < 0.05; Total Effect = −0.0739; ADE = −0.0793), as illustrated in Fig. S7. However, after adjusting for age BMI and gender, this mediation effect was no longer statistically significant.

### Sensitivity analysis

To comprehensively assess the robustness of our primary model, we performed two sets of analyses. First, we performed univariate ± 2 standard deviation perturbation analysis on the selected LR model. The complete results of the univariate perturbation sensitivity analysis are shown in Fig. S8. All curves remained non-overlapping and monotonic within ± 2 standard deviations, indicating that the effects of each predictor on the predicted risk were stable and consistent in direction, which supports the validity of our study results.

Second, to evaluate performance across disease stages, we conducted subgroup analyses. A model trained on the NR (*n* = 437) and BL (*n* = 100) subgroups demonstrated robust performance, with a test set AUC of 0.865 (Table S1). The sample size (*n* = 537) and events per variable (EPV = 11.1, 100 events/9 predictors) basically meet recommended guidelines [43, 44], confirming its validity for predicting early bone loss. In contrast, a model trained on the NR and OP (*n* = 23) subgroups was limited by a small sample size and low EPV (2.6, 23 events/9 predictors). Presenting detailed interpretations from this underpowered analysis could be misleading. Therefore, we focus the interpretation of our predictor set on the primary and NR vs. BL models (Table S1).

## Discussion

### Toward a practical and non-invasive screening tool

With the accelerated aging of the global population, osteoporosis has become a critical public health challenge due to its substantial socioeconomic burden and impact on quality of life [1]. Early identification of high-risk individuals is essential for effective prevention, particularly in high-altitude regions where populations may face unique environmental and lifestyle risk factors. We developed and validated a machine learning-based prediction model for OP&BL in the Tibetan population, integrating traditional risk factors and novel oral microbial biomarkers.

Using elastic net regression for feature selection, we identified nine robust predictors: Age, Gender, BMI, *Abiotrophia*, Frequency of spicy food consumption (H23), Tooth brushing frequency (J5), Frequency of sweet-drink consumption (J3b), Current marital status (Separated/Divorced, A5_3), and Frequency of numbing food consumption (H27). The selection of the λ.1se penalty parameter effectively balanced model complexity and predictive accuracy, reducing the initial 27 features to a final set of nine critical predictors.

Among the five machine learning models evaluated, Logistic Regression (LR) demonstrated the strongest and most stable performance on the test set (AUC = 0.885), along with excellent calibration and the highest net benefit in decision curve analysis. In contrast, more complex ensemble models such as Random Forest and XGBoost showed signs of overfitting. The superior generalizability and interpretability of LR support its potential utility in clinical and public health settings, particularly for smaller cohort studies.

These characteristics make our model a suitable candidate for development as a cost-effective, field-applicable screening approach. Traditional osteoporosis screening relies heavily on DXA, which is often inaccessible in remote high-altitude regions due to cost and logistical constraints. Our model could offer a promising alternative by utilizing non-invasive and easily obtainable variables, including microbial data from saliva samples and simple questionnaire items. This approach could significantly increase feasibility in resource-limited settings. We envision a streamlined workflow: community health workers collect saliva samples and administer a brief questionnaire, while the abundance of key oral microbiota taxa (e.g., *Abiotrophia*) is quantified using rapid quantitative PCR (qPCR) systems. Notably, qPCR testing infrastructure was widely established in China during the COVID-19 pandemic, yet its current utilization rate has declined [45]. This model directly addresses the post-pandemic underutilization of qPCR infrastructure by repurposing it for chronic disease screening 10.3791/69117. It offers a practical solution to elevate the usage rate of existing equipment in remote Chinese clinics for rapid osteoporosis prediction. Furthermore, the ongoing development of handheld, miniaturized point-of-care (POC) qPCR devices [46] indicating a promising pathway toward truly decentralized testing in the future. The collected data could then be processed via a simplified digital application to generate an individual risk score at the POC. This model would serve as an efficient triage system, identifying high-risk individuals for confirmatory DXA scanning, thereby optimizing the use of scarce healthcare resources in remote areas.

### Potential mechanisms underlying the predictive model

The good performance of our model is consistent with the biological and epidemiological plausibility of its key predictors. First, the inclusion of Age, BMI, and Gender as key predictors aligns with their well-established roles in global osteoporosis [47, 48]. Their substantial combined contribution confirms that our model accurately captures these fundamental drivers of bone loss, even in the unique high-altitude setting. This provides a stable clinical anchor for the model. On this basis, the integration of specific oral microbial signatures offers novel insights into high-altitude bone health.

Building on these associations, we explore potential mechanisms linking the identified oral microbial signatures to high-altitude bone health. The unique high-altitude environment, characterized by chronic hypoxia, may influence bone metabolism through several interconnected pathways, which provide a context for interpreting our findings. At altitudes above 3000 m, the partial pressure of oxygen (PO₂) is approximately 65% (495/760 mmHg) of that at sea level, exposing long-term plateau residents, such as Tibetans, to chronic hypoxia [49, 50]. Chronic hypoxia may influence bone metabolism through multiple mechanisms: it can activate hypoxia-inducible factors (HIFs) that regulate bone remodeling and reprogram cellular metabolism to favor glycolysis over mitochondrial oxidative phosphorylation [51]; alter oxidative stress levels that affect osteoblast and osteoclast activity and trigger mitochondrial dysfunction and impaired mitophagy, leading to cell apoptosis and extracellular matrix degradation [52]; and impact nutritional status due to altered dietary patterns, which may subsequently induce gut microbiota dysbiosis and reduce the production of beneficial metabolites like short-chain fatty acids (SCFAs), further exacerbating bone loss [7]. Importantly, growing evidence indicates that the high-altitude environment shapes distinctive oral microbiota compositions [11, 12], which may subsequently influence bone health by modulating systemic inflammation and cellular senescence, as hypoxia can promote the secretion of pro-inflammatory factors that contribute to joint degeneration and bone loss [53, 54].

Building on the above mechanistic insights, our identification of tooth brushing frequency and the microbial genus *Abiotrophia* becomes highly relevant. Infrequent tooth brushing is closely associated with oral dysbiosis [55], the depletion of *Abiotrophia* in the OP&BL group (Fig. 2D) was associated with a protective effect (Fig. 5B), which is consistent with its known biological role. *Abiotrophia* species are nutritionally fastidious microorganisms that require supplemented media for growth. They are considered early colonizers and are part of the normal, healthy oral flora, contributing to oral ecosystem stability [56]. A decrease in *Abiotrophia* abundance is often associated with a dysbiotic shift in the oral microbiome towards a disease-associated state [57]. In our study, the reduced levels of *Abiotrophia* in the OP&BL group may serve as a microbial indicator of a disrupted oral environment, potentially linked to poorer oral health status. This is consistent with our questionnaire data, which showed that self-assessed dental and oral condition (J1) was significantly worse in the OP&BL group (Table 1). Therefore, the selection of *Abiotrophia* as a protective predictor in our model (Fig. 5B) is biologically plausible: its presence may signify a healthier, more stable oral microbiome, which could be associated with lower systemic inflammation levels and a reduced risk of bone loss.

Furthermore, the co-enrichment of *Rothia* and *Veillonella* in the OP&BL suggests a potential synergistic relationship that may contribute to bone loss through nutritional and inflammatory pathways. First, the increased abundance of *Rothia* (Fig. 2C), particularly *R. mucilaginosa*, could potentially affect iron availability. As a producer of the siderophore enterobactin, *Rothia* may sequester iron in the oral environment. This could reduce systemic iron absorption, possibly impairing bone formation as iron serves as a cofactor for collagen cross-linking and osteoblast function [58, 59]. Second, the increase in *Veillonella* (Fig. 2C), particularly *V. parvula*, may promote inflammatory processes linked to bone loss. As shown by Hoare et al., V. parvula can function as an ‘accessory pathogen’ by providing growth factors that support the colonization of periodontopathic bacteria like *Porphyromonas gingivalis* [60]. Although we did not directly measure periodontal pathogens, the elevated *Veillonella* abundance may indicate a shift toward a more inflammatory oral microenvironment. Such changes could trigger pro-inflammatory cytokines (e.g., IL-1β, RANKL) that stimulate osteoclast activity and bone resorption. These two pathways may be interconnected. The iron-sequestering activity of *Rothia* [58, 59] might create conditions that favor bacteria like *V. parvula* and *P. gingivalis*, which can utilize alternative iron acquisition strategies [60]. This potential interaction suggests a hypothetical mechanism whereby *Rothia*-mediated iron restriction could simultaneously impair bone formation and promote an inflammatory oral microbiota, collectively contributing to bone loss.

Additionally, spicy food consumption frequency (H23) was associated with a protective factor (Fig. 5B), which may be related to bioactive compounds (e.g., capsaicin) that are thought to exert anti-inflammatory effects [61, 62]. Although preliminary mediation analysis suggested that *Abiotrophia* might partially mediate the association between spicy food consumption and bone loss, this effect was no longer significant after adjusting for confounders. This indicates that the relationship between diet, microbiota, and bone health is complex and warrants further longitudinal investigation.

### Public health implications, bias considerations, and limitations

Beyond screening, our model offers promising implications for public health and preventive interventions. The inclusion of modifiable factors, such as spicy food consumption and oral hygiene status (inferred from tooth brushing frequency and microbial markers like *Abiotrophia*), provides actionable targets for community-based health programs. This enables a form of precision public health, allowing for targeted interventions, such as dietary advice or oral hygiene education, for high-risk individuals, thereby shifting the paradigm from late-stage diagnosis to proactive, personalized prevention. Improving oral hygiene, modulating diet, and maintaining a balanced oral microbiome may represent practical approaches to support bone health in high-altitude populations [63].

While these implications are promising, several potential sources of bias should be noted when interpreting the findings. First, data on diet, oral hygiene, and lifestyle behaviors were collected via self-reported questionnaires, which are prone to recall and social desirability bias. To mitigate this, all interviews were conducted by trained investigators using standardized instruments and cross-checked for internal consistency. Second, environmental heterogeneity, such as that related to altitude gradients, cultural dietary practices, and seasonal differences, could influence both oral microbiota composition and bone metabolism. Although our study design, which included participants from a defined high-altitude range (> 3,500 m) and a narrow sampling period, aimed to reduce some of this variability, residual environmental effects cannot be completely ruled out and may affect generalizability to other settings.

In addition to these considerations, the study has several limitations. First, the cross-sectional design precludes causal inference regarding the identified predictors and bone loss. Therefore, the associations we report should not be interpreted as causal relationships. Second, the sample size, though sufficient for model development, may limit the generalizability of findings and subgroup analyses, and therefore, external validation in independent cohorts is needed. Third, our participants were Tibetans residing at high altitudes (> 3,500 m) in Southwest China. Although the sampling strategy was designed to be representative of this specific population, the generalizability of our prediction model to other high-altitude populations (e.g., Andean or Ethiopian) or to sea-level populations may be limited due to distinct genetic backgrounds [64], dietary patterns [65], and lifestyle factors [66]. Therefore, the model should not be considered a universal tool for high-altitude osteoporosis screening. Its primary value is as a proof-of-concept for this specific population and as a methodological template. Future studies must validate and likely adapt the model using data from the target population before clinical deployment. Fourth, while 16 S rRNA sequencing provided microbial taxonomic profiles, it has inherent limitations, including restricted taxonomic resolution (primarily to the genus level), inability to detect non-bacterial microorganisms (e.g., fungi, viruses), and lack of direct functional insights. The results are also subject to technical biases from PCR amplification, primer selection, and bioinformatic pipelines, which may affect the accuracy of both taxonomic assignment and relative abundance estimates. Furthermore, this approach lacks direct functional insights. Future metagenomic or metabolomic studies are needed to elucidate the functional mechanisms connecting the oral microbiota and bone metabolism. Fifth, given the cross-sectional design, even a statistically significant mediation effect would not prove causality. The attenuated effect after adjustment further suggests this specific pathway requires cautious interpretation. Future longitudinal studies with functional data are needed to conclusively investigate these interactions.

## Conclusions

In conclusion, this study developed and evaluated a machine learning-based predictive model that integrates oral microbiota data with clinical and lifestyle variables to assess the risk of osteoporosis and bone loss in a high-altitude Tibetan population. The Logistic Regression model demonstrated good predictive performance (AUC = 0.885) and clinical utility in our sample. Our findings highlighted factors, including the oral microbial genus *Abiotrophia* and tooth brushing frequency as significant predictors in this model, suggesting a link between oral ecology and bone health in high-altitude settings. These associations, observed in a cross-sectional study, warrant further longitudinal and mechanistic investigation. If validated externally, the model could represent a step toward a non-invasive, resource-adapted screening tool for such settings. This work illustrates an approach for integrating microbiome data into predictive health models for specific populations.

## Supplementary Information

Additional file 1: Supplementary Materials.

Additional file 2: Table S1. Performance comparison of the primary and sensitivity models on training and test sets. FigS1. Alpha rarefaction curves confirming sequencing depth sufficiency. FigS2. Zero-inflation diagnostics and prevalence filtering of microbial features. Fig. S3. Variance Inflation Factors (VIF) confirming absence of multicollinearity. Fig. S4. Correlation matrix of continuous predictors selected by elastic-net regression. Fig. S5. Confusion matrices of five machine-learning models on the test set. Fig. S6. Ten-fold cross-validation performance of the logistic-regression model. Fig. S7. Exploratory mediation analysis illustrating the indirect effect of *Abiotrophia*. Fig. S8. Sensitivity analysis curves demonstrating stable and monotonic predictor effects.

## Data Availability

The datasets generated and/or analysed during the current study are available in the Genome Sequence Archive (GSA) repository, https://ngdc.cncb.ac.cn/gsa/browse/CRA030554, under the accession number CRA030554.

## Abbreviations

ACME: Average causal mediation effect
ADE: Average direct effect
AUC: Area under the curve
BMD: Bone mineral density
BMI: Body mass index
CMEC: China Multi-Ethnic Cohort
DXA: Dual-energy X-ray absorptiometry
LR: Logistic regression
ML: Machine learning
NB: Naive Bayes
OP: Osteoporosis
OP&BL: Osteoporosis and bone Loss
PCoA: Principal coordinates analysis
QCT: Quantitative computed tomography
QUI: Quantitative ultrasound index
RF: Random forest
SHAP: SHapley additive exPlanations
TE: Total effect
XGB: Extreme gradient boosting
