# Supplementary material for: Machine learning model integrating oral microbiota and clinical features for predicting osteoporosis and bone loss in high-altitude populations
Source: BMC Microbiol. 2026 Mar 5;26:337. doi: 10.1186/s12866-026-04718-0 (PMC13072478; doi:10.1186/s12866-026-04718-0)
Supplement: Supplementary file 1 — Additional file 1: Supplementary Materials. [file 12866_2026_4718_MOESM1_ESM.docx]

**【Individual Questionnaire】**

2024.06

| **Name of Survey Respondent:：**  **Personal Phone：**______________________  **Home Address：**_______ County (City/District)______ Township (Subdistrict)_______ Village (Neighborhood Committee)  (Detailed Address)  **County (City or District) Administrative Code：** □□□□□□  **Township (Subdistrict) Code:** □□  **Village (Residents' Committee) Code:** □□  **Individual Code:：** □□□□ | |
| --- | --- |
| **Investigator's signature：**__________________ | **Date：**________ Year_____ Month______ Day |

**CEMC Project Office**

| Part I General Information **Exposed Section** | |
| --- | --- |
| **A****1 Gender**  □ Male □ Female | |
| **A2 Ethnicity**  □ Han  □ Dong  □ Buyi  □ Yi | □ Miao  □ Bai  □ Tibetan |
| **A3 ID Number**  □□□□□□□□□□□□□□□□□□ | |
| **A4 Your current household registration status is**  □ Agricultural Household Registration □ Non-agricultural Household Registration  □ Unified Resident Household Registration □ No Household Registration | |
| **A5 Your current marital status is**  □ Married/Cohabiting □ Widowed  □ Separated/Divorced □ Never Married | |
| **A6 Your highest level of formal education is** | |
| □ No formal schooling  □ Junior High School  □ College/Technical College | □ Elementary School  □ High School (including vocational/technical schools))  □ University (including graduate studies) |
| **A7 Your occupation type is** | |
| □Agriculture, forestry, animal husbandry, or fishery worker | **□**  Retired/on leave ••••A7a |
| □ Factory worker  □ Administrative or managerial staff  □ Professional/technical personnel (doctor, teacher, scientist/engineer)  □ Sales or service worker | □ Homemaker  □ Self-employed business owner  □ Unemployed/laid off  □ Other or difficult to classify |
| **A7a Your last occupation before retirement was** | |
| □ Agriculture, forestry, animal husbandry, or fishery worker | □ Homemaker |
| □ Factory worker | □ Self-employed business owner |
| □ Administrative or managerial staff | □ Unemployed/laid off |
| □ Professional or technical personnel (doctors, teachers, scientists) | □ Other or difficult to classify |
| □ Sales or service worker | |
| **A8**  **A8a Do you participate in any of the following medical insurance programs?** | |
| □ Urban Employee Basic Medical Insurance (including fully government-funded medical insurance) | |
| □ Urban Resident Basic Medical Insurance | |
| □ New Rural Cooperative Medical Scheme | |
| □ Urban and Rural Resident Basic Medical Insurance | |
| □ None of the above medical insurance | |
| **A8b Have you purchased commercial medical insurance?** □ Yes □ No | |
| **A9 Including yourself, how many people live together in your household?** ____________ people. | |
| **A10 Primary Family Contact**  A10a Name：___________________  A10b Relationship to you: □ Father □ Mother □ Child □ Spouse □ Other___________  A10c Contact Number ：_______________ | |
| **A13 What was your household's total annual income in the past year (before taxes)?**  □ <12000 RMB  □ 12000-19999 RMB  □ 20000-59999 RMB  □ 60000-99999 RMB  □ 100000-199999 RMB  □ ≥200000 RMB | |
| **A16 Does your household possess any of the following items?**  Yes No  □ □ Owned house/apartment  □ □ Running water  □ □ Private bathroom  □ □ Landline or mobile phone  □ □ Automobile  □ □ Motorcycle/motor vehicle  □ □ Computer (including laptop, desktop, tablet, etc.)  □ □ Internet access (wireless or broadband, etc.)  □ □ Email/WeChat/Weibo/QQ (owned by respondent)  □ □ Traveled outside the country in the past five years | |

| **Part II: Smoking Status and Exposure to Personal Environmental Factors** | | |
| --- | --- | --- |
| **B1 Do you smoke?** | | |
| □ No ••••🡺 | | **B8** |
| □ Yes (have smoked over 100 cigarettes total）••••🡺 | | **B4** |
| □ Quit (abstained for 6+ months) | |  |
| **B2 How long have you been smoke-free? (continuous abstinence for 6+ months)** □□years | | |
| **B2a What were your reasons for quitting? (multiple selections)**  □ Already suffering from certain illnesses  □ Concerned about developing certain illnesses  □ Financial reasons  □ Family opposition  □ Other | | |
| **B4 When you first developed the smoking habit, what type of tobacco did you primarily use?**  □ Cigarettes  □ Other tobacco products (e.g., pipe tobacco, hookah, pipe tobacco, hand-rolled cigarettes, etc.)  □ Mixed use of cigarettes and other tobacco products  □ E-cigarettes | | |
| **B5 How long after waking up in the morning did you smoke your first cigarette?**  □ >60 minutes □ 31～60 minutes  □ 6～30 minutes □ ≤5 minutes | | |
| **B6 What type(s) of tobacco did you typically smoke currently or before quitting, and how much? (multiple selections allowed)** | | |
| □ Cigarettes with filters | □□□sticks/week | |
| □ Cigarettes without filters | □□□sticks/week | |
| □Hand-rolled cigarettes/pipe tobacco | □□□packs/week | |
| □ Snuff | □□□packs/week | |
| □ Pipe tobacco/hookah | □□□packs/week | |
| □ Cigars | □□□packs/week | |
| □ E-cigarettes | □□□packs/week | |
| **B7 When smoking, where do you typically draw the smoke?** | | |
| □ Draw into mouth and exhale ••••🡺 | **B8** | |
| □ Draw into throa ••••🡺 | **B8** | |
| □ Draw deeply into lungs ••••🡺 | **B7a** Since starting to smoke, have you consistently inhaled deeply into your lungs?  □Yes □No | |
| **B8 Have you been exposed to secondhand smoke from cigarettes or other tobacco products at home, work, or recreational venues in the past?** | | |
| □ Yes ••••🡺 | **B8a** Duration □□□hourss/week，accumulated over □□□years | |
| □ No |  | |
| **B9 Do you frequently use or come into contact with pesticides in your daily life or work?**  □ Yes, duration of exposure: □□years  □ No | | |
| **B23 Have you moved in the past 5 years?** | | |
| □ Yes | | |
| □ No ••••🡺 **C2** | | |
| **B24 Your previous residential address was** | | |

| Part III: Alcohol Consumption | | | | | |
| --- | --- | --- | --- | --- | --- |
| C2 Over the past year, approximately how often did you drink alcohol? | | | | | |
| □ Never or almost never drank alcoho | | | | |  |
| □ Only occasionally on special occasions (e.g., celebrations or holidays)  □ Only drank during specific months of the year (e.g., busy farming seasons or summer), generally abstaining during other seasons  □ Drink monthly throughout the year regardless of season, but less than weekly | | | | |  |
| □Drink at least weekly throughout the year regardless of season ••••🡺 | | | | | **C4** |
| C3  C3a Please recall: Have you ever had a period where you drank weekly for at least one year? | | | | | |
| □ Yes ••••🡺 | | | | | **C3b** |
| □ No ••••🡺 | | | | | **D1** |
| C3b How long has it been since you stopped the above situation (drinking weekly for a year)?  □□years ••••🡺 D1 | | | | | |
| C4 Over the past year, on average, how many days per week did you drink?  □ 1-2 days/week  □ 3-5 days/week  □ Daily or nearly daily | | | | | |
| C5 At approximately what age did you begin developing the habit of drinking weekly? □□years old | | | | | |
| C6 Below, please indicate your typical daily alcohol consumption in three different scenarios (if mixing drinks in special situations, select up to three types simultaneously): | | | | | |
|  | Under normal circumstances  (Select one) | |  |  | |
| Beer | □□bottles/week | |  |  | |
| Yellow wine/rice wine/fermented rice wine/fruit wine (<10°) | □□liang/week | |  |  | |
| Barley wine | □□liang/week | |  |  | |
| Wine | □□liang/week | |  |  | |
| High-proof baijiu or blended liquor (≥40°) | □□liang/week | |  |  | |
| Low-proof baijiu (<40°) | □□liang/week | |  |  | |
| High-proof rice wine (30°-40°) | □□liang/week | |  |  | |
| Rice wine（10°-29.9°） | □□liang/week | |  |  | |
| C7 When do you typically drink alcohol during the day?  □ Usually before meals  □ Usually during meals  □ Usually after meals or between meals  □ Usually before bedtime  □ Occurs in various situations | | | | | |
| C8 Do you experience flushing, rapid heartbeat, or dizziness after drinking?  □ Occurs after one or two sips  □ Occurs after small amounts (one or two liang or less)  □ Occurs only after heavy drinking  □ No such reactions occur | | | | | |
| C9 In the past month, have you experienced any of the following alcohol-related situations? (Multiple selections allowed) | | | | | |
|  | | Yes No | | | |
| Unable to perform tasks due to excessive drinking | | □ □ | | | |
| Feel depressed, impulsive, or unable to control oneself after drinking | | □ □ | | | |
| Addicted to drinking, unable to stop | | □ □ | | | |
| Experienced symptoms like trembling after stopping drinking | | □ □ | | | |
| C10 Has there been a noticeable change in your alcohol consumption compared to several years ago?  □ Not much change  □ Significantly increased compared to previous years  □ Significantly decreased compared to previous years | | | | | |

| Part IV Tea and Beverage Consumption | | | |
| --- | --- | --- | --- |
| 1. **Tea Consumption** | | | |
| **D1 Have you ever consumed tea weekly for over six consecutive months?** | | | |
| □ Yes | | | |
| □ No ••••🡺  **D9** | | | |
| **D2 At approximately what age did you develop the habit of drinking tea?**  □□years old | | | |
| **D3 Over the past year, on average, how many days per week did you drink tea?**  □ I no longer drink tea  **••••🡺 D3a** □□years old when I stopped drinking tea | | | |
| □ 1-2 days/week | | | |
| □ 3-5 days/week | | | |
| □ Every day or almost every day | | | |
| **D4 When you drink tea, which type do you most commonly consume? How many cups do you typically drink per day? (Please select the most frequently consumed type of tea)** | | | |
| Green Tea | □□cups/day | | |
| Flower tea | □□cups/day | | |
| Black tea (brick tea, Pu'er tea) | □□cups/day | | |
| Sweet tea (milk + black tea) | □□cups/day | | |
| Black tea | □□cups/day | | |
| Oolong tea (Tieguanyin) | □□cups/day | | |
| Buckwheat tea | □□cups/day | | |
| White tea (Silver Needle) | □□cups/day | | |
| **D5 When drinking tea, how many times per day do you typically replace the tea leaves? (Enter 0 if none)** | | □□times/day | |
| **D6 When brewing tea, approximately how many grams of tea leaves do you use per serving? (One mineral water cap holds about 2g)** | | □□grams/serving | |
| **D7 Do you generally prefer light, medium, or strong tea?**  □ Light tea □ Medium □ Strong tea | | | |
| **D8 Do you usually prefer scalding hot tea, hot tea, or warm/cool tea?**  □ Scalding hot tea □ Hot tea □ Warm/cool tea | | | |
| 1. **Beverages** | | | |
| **D9 Have you ever consumed beverages weekly for over six consecutive months?** | | | |
| □ Yes | | | |
| □ No ••••🡺 **E1** | | | |
| **D10 At approximately what age did you start drinking beverages regularly?** □□years old | | | |
| **D11 Over the past year, on average, how many days per week did you drink beverages?**  □I no longer drink beverages ••••🡺  **D11a**  □□years old stopped drinking beverages | | | |
| □ 1-2 days/week  □ 3-5 days/week  □ Daily or nearly daily | | | |
| **D12 When you consume beverages, which type do you drink most frequently? How many servings per week? (Select the most commonly consumed beverage)** | | | |
| Additive-containing beverages (carbonated drinks, dairy-based drinks, sports drinks, plant-based protein drinks, tea beverages) | | | □□servings/week |
| Coffee and coffee beverages | | | □□servings/week |
| Other beverages | | | □□servings/week |

| Part V Individual and Family Health Status | | | | | |
| --- | --- | --- | --- | --- | --- |
| **E1** |  |  |  |  |  |
|  | None | Mild | Moderate | Severe | Unable to complete  /Very severe |
| E1a1 Today, did you have difficulty moving around: | □ | □ | □ | □ | □ |
| E1a2 Today, did you have difficulty with self-care (bathing or dressing): | □ | □ | □ | □ | □ |
| E1a3 Do you have difficulty engaging in daily activities (such as work, study, housework, family, or leisure activities) today: | □ | □ | □ | □ | □ |
| E1a4 Do you have pain or discomfort today: | □ | □ | □ | □ | □ |
| E1a5 Do you have anxiety or depression today: | □ | □ | □ | □ | □ |
| E1b The scale on the right ranges from 0 to 100, where 100 represents the best health you could imagine and 0 represents the worst health you could imagine. Where would you place your health today on this scale? \| | \|---\|---\|---\|---\|---\|---\|---\|---\|---\|---\|  0 10 20 30 40 50 60 70 80 90 100  Worst possible health Best possible health  __________ points | | | | |
| **E2 How would you rate your current health?**  □ Very good  □ Fairly good  □ Average  □ Fairly poor  □ Very poor | | | | | |
| **E4 How often do you typically have a bowel movement?**  □ More than once daily  □ Roughly once daily  □ Roughly every other day  □ Less than 3 times weekly | | | | | |
| **E5 When brushing your teeth, do your gums bleed?**  □ Rarely or never bleed  □ Sometimes bleed  □ Often bleed | | | | | |
| **E8 Is your biological mother still alive?** | | | | | |
| □ Yes ••••🡺 **E8a** Current age:：□□□ | | | | | |
| □ No ••••🡺 **E8b** Age at death：□□□ | | | | | |
| □ Unknown | | | | | |
| **E9 Is your biological father still alive?** | | | | | |
| □ Yes ••••🡺  **E9a** Current age：□□□ | | | | | |
| □ No ••••🡺 **E9b** Age at death：□□□ | | | | | |
| □ Unknown | | | | | |

| Part VI Physical Activity |
| --- |
| **Screening Question: Have you engaged in agricultural or pastoral work in the past year?**  □ Yes------Answer only “2. Physical Activity - Agricultural/Pastoral Workers” and “3. Physical Activity - Common Section”  □ No------Answer only “1. Physical Activity - Non-Agricultural/Pastoral Workers” and “3. Physical Activity - Common Section” |
| 1. **Physical Activity - Non-Agricultural Workers** |
| **F1 Over the past year, did your work primarily involve sitting or standing, or did it involve physical labor?** |
| □ Primarily sitting (e.g., administrative staff, secretaries, etc.) ••••🡺 **F1a** Average weekly sitting time: □□□hourss |
| □ Primarily standing (e.g., sales clerks, security guards, etc.) |
| □ Primarily general physical labor (easily sweating in normal temperatures, e.g., plumbers, electricians, carpenters, bricklayers, etc.) |
| □ Primarily heavy physical labor (easily sweating in normal temperatures, e.g., loading/unloading, mining, steel production) |
| □ Retired, engaged in household duties, unemployed for over a year, or physically disabled and unable to work normally ••••🡺 **F13** |
| **F2 How many hours per week do you typically work?**  □□□hours |
| **F3 Over the past year, what was your primary mode of transportation to and from work or other activities? (Single choice)** |
| □ Walking  □ Riding a motorcycle/electric scooter  □ Riding a bicycle  □ Private or public transportation (car, subway, ferry) |
| □ Usually working at or near home ••••🡺 **F13** |
| **F4 How long do you typically spend commuting to and from work (or work-related outings) each day?** □□□minutes |
| 1. **Physical Activity Status - Agricultural and Pastoral Workers** |
| **F5 Does your production activity have distinct busy and idle periods?** |
| □ Yes |
| □ No ••••🡺  **F7** |
| **F6** |
| **F6a During the past year's busy farming season, how many months did the cumulative busy period last?** □□months |
| **F6b During the peak farming season in the past year, what was your primary method of labor?**  □ Primarily manual labor  □ Semi-mechanized  □ Primarily mechanized |
| **F6c During the peak farming season in the past year, how many hours per day did you typically spend on farm work?**  □□.□hours |
| **F7 During non-busy periods or under normal circumstances, how many hours per week do you typically spend on farm work?** □□□hours/week |
| **F8 Besides farm work, do you usually engage in other jobs simultaneously?** |
| □ Yes |
| □ No ••••🡺 **F11** |
| **F9 For the other jobs you do, is the work primarily seated or standing, or does it involve heavy physical labor?** |
| □Primarily seated (e.g., weaving, sewing) ••••🡺 **F9a** Average weekly seated hours: □□□hours |
| □ Primarily standing (e.g., sales clerk, security guard) |
| □ Primarily general physical labor (carpentry, electrical work, construction, etc.) |
| □ Primarily heavy physical labor (porters, mining, loading/unloading, etc.) |
| **F10 Excluding farm work, how many hours per week do you typically spend on other work?** □□□hours |
| **F11 In the past year, what was your primary mode of transportation to work? (Single choice)** |
| □ Walking |
| □ Motorcycle/electric scooter |
| □ Bicycle |
| □ Private or public transportation (car, subway, ferry) |
| □ Usually near home ••••🡺 **F13** |
| **F12 How long do you typically spend commuting to and from work (or other labor activities) each day?** □□□minutes |
| 1. **Physical Activity - Common Section** |
| **F13 How often did you engage in physical exercise during your free time over the past year?** |
| □ Never or almost never ••••🡺 **F17** |
| □ 1-3 times per month ••••🡺  **F17** |
| □ 1-2 times per week |
| □ 3-5 times per week |
| □ Daily or almost daily |
| **F14 If you exercise weekly, which activity do you most frequently engage in?**  □ Tai Chi/Qigong/Walking  □ Brisk walking/Health exercises/Yangko dance/Square dancing  □ Running/Aerobics  □ Swimming  □ Ball sports (basketball, table tennis, badminton, etc.)  □ Weight training  □ Other (e.g., mountain climbing) |
| **F15 Over the past year, how many cumulative hours per week did you spend on recreational physical activities?** □□□hours/week |
| **F17 On average, how many cumulative hours per week do you engage in vigorous physical activities?** □□□hours/week |
| **F18 On average, how many cumulative hours per week do you spend on various household chores (including childcare)?** □□□hours/week |
| **F19 How many hours per week do you typically spend sitting during leisure time (e.g., playing board/card games, using mobile/tablet devices, watching TV, reading, knitting)?** □□□hours/week |
| **F20 Compared to previous years, has your weight changed significantly over the past year?**  □ No significant change  □ Gained at least 5 jin (approx. 2.5 kg)  □ Lost at least 5 jin (approx. 2.5 kg) |

| Part VII Female Reproductive and Fertility History (For Females Only) **If Male •••••••••••••••••••••••••****•••••••••••••••••••••🡺Skip to Part VIII** |
| --- |
| **G2 What is your current menstrual status?** |
| □ Regular menstruation ••••🡺 **G6** |
| □ Irregular menstruation ••••🡺 **G6** |
| □ Perimenopausal ••••🡺  **G6** |
| □ Postmenopausal (no menstruation for ≥12 months) |
| □ Currently pregnant ••••🡺  **G6** |
| **G2a If postmenopausal, age at menopause:**  □□years old |
| **G6 Have you ever taken birth control pills?** |
| □ Never used ••••🡺 **G8** |
| □ Yes ••••🡺 **G6a** Total duration of long-acting or short-acting contraceptive use: □□years  **G6b** Total number of emergency contraceptive pills used: □□times |
| **G8 Have you undergone a hysterectomy?** |
| □ Yes ••••🡺 **G8a** Age at time of surgery: □□years old |
| □ No |
| **G9 Have you undergone ovarian removal (unilateral or bilateral)?** |
| □ Yes ••••🡺 **G9a** Age at surgery: □□years old  ••••🡺 **G9b** □Unilateral □Bilateral |
| □ No |
| **G12 Were you diagnosed with any of the following conditions during pregnancy?** |
| Gestational diabetes mellitus □ Yes □ No |
| Preeclampsia/Eclampsia □ Yes □ No |
| Gestational hypertension □ Yes □ No |
| **G13 Have you ever undergone hormone replacement therapy?** |
| □ Yes ••••🡺  **G13a** Duration of treatmen □ years |
| □ No |
| □ Unknow |

| Part VIII: Dietary Conditions | | | | | | | | | | |
| --- | --- | --- | --- | --- | --- | --- | --- | --- | --- | --- |
| **H1** | | | | | | | | | | |
| H1a How many people in your household usually eat breakfast at home in the past month? □□people | | | | | | | | | | |
| H1b How many people in your household usually eat lunch at home in the past month? □□people | | | | | | | | | | |
| H1c How many people in your household usually eat dinner at home in the past month? □□people | | | | | | | | | | |
| **H2 What cooking oils does your household consume most frequently? (Select up to 2) (1 kg = 2 jin)** | | | | | | | | | | |
| □Rapeseed oil/sesame oil _______ jin/month | | | | | | | | | | |
| □ Peanut oil _______ jin/month | | | | | | | | | | |
| □ Soybean oil _______ jin/month | | | | | | | | | | |
| □ Lard _______ jin/month | | | | | | | | | | |
| □ Blended oil _______ jin/month | | | | | | | | | | |
| □ Other oils _______ jin/month | | | | | | | | | | |
| **H3 How much salt does your household consume on average each month?**  □□□□grams/month (1 jin = 500 grams) | | | | | | | | | | |
| **Please recall whether you typically consumed the following foods over the past year, estimating their weight (either “raw weight” or “cooked weight”) and frequency of consumption. This survey concerns individual dietary habits!** | | | | | | | | | | |
|  | | a Yes/No  consumed | b Frequency of consumption  (select only one option) | | | | | | c Per serving (grams) (1 liang = 50 grams) | |
|  |  | Yes No | b1  Times /day | | | b2  Times /week | b3  Times /month | b4  Times /year | Raw weight | Cooked weight |
| **H4** | Rice | □ □ | □ | | | □ | □ | □ | — | □g |
| **H5** | Noodles | □ □ | □ | | | □ | □ | □ | — | □g |
| **H6** | Whole grains (corn, sorghum, oats, etc.) | □ □ | □ | | | □ | □ | □ | □g | — |
| **H7** | Starchy tubers (sweet potatoes, potatoes) | □ □ | □ | | | □ | □ | □ | □g | — |
| **H8** | Red meat and processed red meat products | □ □ | □ | | | □ | □ | □ | □g | — |
| **H9** | Poultry and poultry products | □ □ | □ | | | □ | □ | □ | □g | — |
| **H10** | Aquatic/seafood products | □ □ | □ | | | □ | □ | □ | □g | — |
| **H11** | Eggs and egg products | □ □ | □ | | | □ | □ | □ | □g | — |
| **H12** | Fresh vegetables | □ □ | □ | | | □ | □ | □ | □g | — |
| **H13** | Soy products (including soy milk) | □ □ | □ | | | □ | □ | □ | □g | — |
| **H14** | Pickled vegetables | □ □ | □ | | | □ | □ | □ | □g | — |
| **H15** | Fresh fruits | □ □ | □ | | | □ | □ | □ | □g | — |
| **H16** | Dairy and dairy products (e.g., milk, yogurt) | □ □ | □ | | | □ | □ | □ | □g | — |
| **H16a** | Nuts | □ □ | □ | | | □ | □ | □ | □g | — |
|  | | a Yes/No  consumed | b Frequency of consumption  (select only one option) | | | | | | c Per serving (grams) (1 liang = 50 grams) | |
|  |  | Yes No | b1  Times /day | | | b2  Times /week | b3  Times /month | b4  Times /year | Raw weight | Cooked weight |
| **HH**  **Tibetan** | HH1 Tsampa | □ □ | □ | | | □ | □ | □ | — | □g |
|  | HH2 Tibetan Noodles | □ □ | □ | | | □ | □ | □ | — | □g |
|  | HH3 Butter Tea | □ □ | □ | | | □ | □ | □ | — | □g |
|  | HH4 Plain Tea | □ □ | □ | | | □ | □ | □ | — | □g |
|  | HH5 Raw Beef | □ □ | □ | | | □ | □ | □ | □g | — |
| **HN**  **Highland**  **Han Chinese** | HN1 Barley Flour | □ □ | □ | | | □ | □ | □ | — | □g |
|  | HN2 Ham/Smoked Pork | □ □ | □ | | | □ | □ | □ | □g | — |
|  | HN3 Oil Tea | □ □ | □ | | | □ | □ | □ | — | □g |
|  | HN4 Wild Mushrooms | □ □ | □ | | | □ | □ | □ | □g | — |
| **HM**  **Yi**  **Bai** | HM1 Ham/Smoked Pork | □ □ | □ | | | □ | □ | □ | □g | — |
|  | HM2 Pig Liver | □ □ | □ | | | □ | □ | □ | □g | — |
|  | HM3 Raw Hide/Raw Meat | □ □ | □ | | | □ | □ | □ | □g | — |
|  | HM4 Wild Mushrooms | □ □ | □ | | | □ | □ | □ | □g | — |
|  | HM5 Milk Cake | □ □ | □ | | | □ | □ | □ | □g | — |
|  | HM6 Milk Curd | □ □ | □ | | | □ | □ | □ | □g | — |
| **HG**  **Miao**  **Dong**  **BuYei** | HG1 Glutinous Rice and Derivatives | □ □ | □ | | | □ | □ | □ | □g | — |
|  | HG2 Pickled Fish/Meat | □ □ | □ | | | □ | □ | □ | □g | — |
|  | HG3 Dried/Smoked Meat | □ □ | □ | | | □ | □ | □ | □g | — |
|  | HG4 Sour Soup (White Sour) | □ □ | □ | | | □ | □ | □ | — | — |
|  | HG5 Sour Soup (Red Sour) | □ □ | □ | | | □ | □ | □ | — | — |
|  | HG6 Fermented Beef/Mutton | □ □ | □ | | | □ | □ | □ | — | — |
|  | HG7 Purple Blood Meat | □ □ | □ | | | □ | □ | □ | — | — |
|  | HG8 Fish Grass | □ □ | □ | | | □ | □ | □ | □g | — |
|  | HG9 Bracken | □ □ | □ | | | □ | □ | □ | □g | — |
|  | HG10 Oil Tea | □ □ | □ | | | □ | □ | □ | — | — |
| \|  \| a Yes/No  consumed \| b Frequency of consumption  (select only one option) \| \| \| \| c Per serving (grams) (1 liang = 50 grams) \| \| \| \| --- \| --- \| --- \| --- \| --- \| --- \| --- \| --- \| --- \| \| Yes No \| b1  Times /day \| b1  Times /day \| b1  Times /day \| b1  Times /day \| Raw weight \| Cooked weight \| \| \| \| **HF**  **Han Chinese in the Chongqing region** \| HF1 Small pointed chili peppers (fresh, red, green) \| □ □ \| □ \| □ \| □ \| □ \| □g \| — \|  \| — \|  \|  \|  \|  \|  \|  \|  \|  \|  \|  \|  \| \| --- \| --- \| --- \| --- \| --- \| --- \| --- \| --- \| --- \| --- \| --- \| --- \| --- \| --- \| --- \| --- \| --- \| --- \| --- \| --- \| --- \| --- \| \| HF2 Dried red chili peppers \| □ □ \| □ \| □ \| □ \| □ \| □g \| — \|  \| — \|  \|  \|  \|  \|  \|  \|  \|  \|  \|  \|  \| \| HF3 Chili peppers (small, red, green) \| □ □ \| □ \| □ \| □ \| □ \| □g \| — \|  \| — \|  \|  \|  \|  \|  \|  \|  \|  \|  \|  \|  \| \| HF4 Bell peppers (green, red, yellow, purple, white, etc.) \| □ □ \| □ \| □ \| □ \| □ \| □g \| — \|  \| — \|  \|  \|  \|  \|  \|  \|  \|  \|  \|  \|  \| \| HF5 Pickled chili peppers \| □ □ \| □ \| □ \| □ \| □ \| □g \| — \|  \| — \|  \|  \|  \|  \|  \|  \|  \|  \|  \|  \|  \| \| HF6 Chili oil \| □ □ \| □ \| □ \| □ \| □ \| □g \| — \|  \| — \|  \|  \|  \|  \|  \|  \|  \|  \|  \|  \|  \| \| HF7 Chili sauce \| □ □ \| □ \| □ \| □ \| □ \| □g \| — \|  \| — \|  \|  \|  \|  \|  \|  \|  \|  \|  \|  \|  \| \| HF8 Chili powder \| □ □ \| □ \| □ \| □ \| □ \| □g \| — \|  \| — \|  \|  \|  \|  \|  \|  \|  \|  \|  \|  \|  \| \| HF9 Spicy hot pot \| □ □ \| □ \| □ \| □ \| □ \| — \| — \|  \| — \|  \|  \|  \|  \|  \|  \|  \|  \|  \|  \|  \| \| \| \| \| \| \| \| \| | | | | | | | | | | |
| **H17 In the past year, have you taken any nutritional supplements for at least one month?** | | | | | | | | | | |
| □ Yes | | | | | | | | | | |
| □ No ••••🡺  **H23** | | | | | | | | | | |
| **H18 Types of nutritional supplements taken in the past year** | | | | | | | | | | |
| H18a Fish oil/cod liver oil | | | | | □ Yes □ No | | | |  | |
| H18b Vitamin D | | | | | □ Yes □ No | | | |  | |
| H18c Other vitamins (excluding vitamin D) | | | | | □ Yes □ No | | | |  | |
| H18d Calcium supplements | | | | | □ Yes □ No | | | |  | |
| H18e Other health supplements,  please specify__________ | | | | □ Yes □ No | | | | |  | |
| **H23 Over the past month, approximately how often did you eat spicy foods?** | | | | | | | | | | |
| □ Never/Rarely ••••🡺 **H27** | | | | | | | | | | |
| □ few times, but less than weekly ••••🡺 **H27** | | | | | | | | | | |
| □ 1-2 days per week | | | | | | | | | | |
| □ 3-5 days per week | | | | | | | | | | |
| □ Daily or almost daily | | | | | | | | | | |
| **H25 Do you generally prefer mildly spicy, moderately spicy, or extremely spicy foods?** | | | | | | | | | | |
| □ Mildly spicy | | | | | | | | | | |
| □ Moderately spicy | | | | | | | | | | |
| □ Extremely spicy | | | | | | | | | | |
| **H26 When eating spicy food, which of these spicy ingredients do you typically use? (Multiple selections allowed)** | | | | | | | | | | |
| □ Chili sauce | | | | | | | | | | |
| □ Chili oil | | | | | | | | | | |
| □ Dried red chili peppers | | | | | | | | | | |
| □ Fresh chili peppers (small, red/green) | | | | | | | | | | |
| □ Fresh green peppers and bell peppers (green, red, yellow, purple, white, etc.) | | | | | | | | | | |
| □ Other (e.g., curry or spicy spices) | | | | | | | | | | |
| **H27 How often did you eat Sichuan peppercorn-infused foods (containing Sichuan peppercorns) in the past month?** | | | | | | | | | | |
| □ Never/Rarely ••••🡺 **H31** | | | | | | | | | | |
| □ few times, but less than weekly ••••🡺 **H31** | | | | | | | | | | |
| □ 1-2 days per week | | | | | | | | | | |
| □ 3-5 days per week | | | | | | | | | | |
| □ Daily or almost daily | | | | | | | | | | |
| **H29 Do you generally prefer mildly numbing, moderately numbing, or extremely numbing foods?**  □ Mildly numbing □ Moderately numbing □ Extremely numbing | | | | | | | | | | |
| **H30 When eating Sichuan-style dishes (containing Sichuan peppercorns), which of the following Sichuan peppercorn ingredients do you typically use? (Multiple selections allowed)** | | | | | | | | | | |
| □ Sichuan peppercorn oil | | | | | | | | | | |
| □ Dried Sichuan peppercorns | | | | | | | | | | |
| □ Fresh Sichuan peppercorns | | | | | | | | | | |
| □ Other (spices containing Sichuan peppercorns)  **H31 In the past year, have you engaged in any of the following behaviors?**   \|  \| Daily \| 4-6 days per week \| 1-3 days per week \| Once per month \| Never or very rarely \| \| --- \| --- \| --- \| --- \| --- \| --- \| \| Skipping breakfast \| □ \| □ \| □ \| □ \| □ \| \| Eating at street stalls (including late-night snacks, street food, grilled items, etc.) \| □ \| □ \| □ \| □ \| □ \| \| Dining at restaurants \| □ \| □ \| □ \| □ \| □ \| \| Eating takeout food \| □ \| □ \| □ \| □ \| □ \| \| Eating fried foods (e.g., fried dough sticks, fried chicken) \| □ \| □ \| □ \| □ \| □ \| \| Eating Western fast food (e.g., pizza/hamburgers) \| □ \| □ \| □ \| □ \| □ \| | | | | | | | | | | |

| Part IX: Life Events, Social Support, and Psychological Well-being | | | | |
| --- | --- | --- | --- | --- |
| 1. **Life Satisfaction** | | | | |
| **I1 Are you satisfied with your current life situation?** | | | | |
| □ Very satisfied | | | | |
| □ Generally satisfied | | | | |
| □ Neither satisfied nor dissatisfied | | | | |
| □ Dissatisfied | | | | |
| □ Very dissatisfied | | | | |
| 1. **Sleep** | | | | |
| **I2 Please answer the following questions based on your actual situation over the past month (excluding special circumstances like travel or jet lag):** | | | | |
| I2a. Over the past month, what time did you usually go to bed at night (24-hour clock)? □□:□□ | | | | |
| I2b. Over the past month, how many minutes did it usually take you to fall asleep after going to bed? □□□ minutes | | | | |
| I2c. In the past month, what time did you usually wake up in the morning (24-hour clock)? □□:□□ | | | | |
| I2d. In the past month, how many hours did you usually sleep each night (not equal to time spent in bed)? □□.□ hours | | | | |
| **I2e. In the past month, were you troubled by sleep problems due to the following situations：** | | | | |
| \|  \| None \| <1times/week \| 1-2times/week \| ≥3times/week \| \| --- \| --- \| --- \| --- \| --- \| \| I2e_1 Difficulty falling asleep (unable to sleep within 30 minutes) \| □ \| □ \| □ \| □ \| \| I2e_2 Frequent nighttime awakenings or early morning awakenings \| □ \| □ \| □ \| □ \| \| I2e_3 Nighttime bathroom visits \| □ \| □ \| □ \| □ \| \| I2e_4 Difficulty breathing \| □ \| □ \| □ \| □ \| \| I2e_5 Coughing or loud snoring \| □ \| □ \| □ \| □ \| \| I2e_6 Feeling cold \| □ \| □ \| □ \| □ \| \| I2e_7 Feeling hot \| □ \| □ \| □ \| □ \| \| I2e_8 Nightmares \| □ \| □ \| □ \| □ \| \| I2e_9 Pain or discomfort \| □ \| □ \| □ \| □ \| \| I2e_10 Other sleep-disrupting factors \| □ \| □ \| □ \| □ \| | | | | |
| I2f **Overall, how would you rate your sleep quality in the past month?**  □ Excellent □ Fairly good □ Fairly poor □ Very poor | | | | |
| **I2g. In the past month, how often did you use medication to induce sleep?**  □ None □ ＜１times／week □１～２times／week □ ≥３times／week | | | | |
| **I2h. In the past month, have you often felt sleepy?**  □ None □ ＜１times／week □１～２times／week □ ≥３times／week | | | | |
| **I2i. In the past month, have you felt lacking in energy for activities?**  □ No □ Occasionally □ Sometimes □ Frequently  **I3e** **How long have you experienced the above sleep issues?** _____years | | | | |
| **I4 Do you have a habit of taking afternoon naps?** | | | | |
| □ Yes | | | | |
| □ No ••••🡺  **I8** | | | | |
| **I5 On average, how many minutes do you typically nap each day?**  □□□minutes | | | | |
| 1. **Psychological Condition** | | | | |
| **I8 How often have you experienced the following symptoms in your life over the past two weeks?** | | | | |
|  | Not at all | A few days | More than half the days | Almost every day |
| I8a Lack of energy or interest in activities | □ | □ | □ | □ |
| I8b Feeling down, depressed, or hopeless | □ | □ | □ | □ |
| **I9** Over the past two weeks, how much time have you been troubled by the following issues? | | | | |
|  | Not at all | A few days | More than half the days | Almost every day |
| I9a Feeling tense, anxious, or irritable | □ | □ | □ | □ |
| I9b Unable to stop or control worrying | □ | □ | □ | □ |
| **4. Mini Mental State Exam** | | | | |
| **Triage Question: What is your age?** | | | | |
| □ **<60years old** ••••🡺 **Outcome Section** | | | | |
| □ **≥60years old** | | | | |
| **I10 What year is it?** | | | | |
| □ Correct □ Incorrect □ Not Assessed | | | | |
| **I11 What season is it now?** | | | | |
| □ Correct □ Incorrect □ Not Assessed | | | | |
| **I12 What date is it today?** | | | | |
| □ Correct □ Incorrect □ Not Assessed | | | | |
| **I13 What day of the week is it today?** | | | | |
| □ Correct □ Incorrect □ Not Assessed | | | | |
| **I14 What month is it now?** | | | | |
| □ Correct □ Incorrect □ Not Assessed | | | | |
| **I15 Which province are we in now?** | | | | |
| □ Correct □ Incorrect □ Not Assessed | | | | |
| **I16 Which city (or state, region, county) are we in now?** | | | | |
| □ Correct □ Incorrect □ Not Assessed | | | | |
| **I17 Which district (town/village) are we in now?** | | | | |
| □ Correct □ Incorrect □ Not Assessed | | | | |
| **I18 Which floor are we on now?** | | | | |
| □ Correct □ Incorrect □ Not Assessed | | | | |
| **I19 What is this place called?** | | | | |
| □ Correct □ Incorrect □ Not Assessed | | | | |
| **I20 How would you rate your current memory? Excellent, very good, good, average, or poor?** | | | | |
| □ Excellent □ Very good □ Good  □ Average □ Poor | | | | |
| **I21** **I will now name three objects. After I finish, please repeat them. Ball, flag, tree. Please repeat these three objects for me.** | | | | |
| □ Ball □ Flag □ Tree  □ None of the above □ Not assessed | | | | |
| **I22 100 minus 7, subtract 5 times consecutively.** | | | | |
| **I22a** **Record answer** ______ | | | | |
| □ Don't know/Refuse to answer ••••🡺 **I23** | | | | |
| **I22b Record answer** ______ | | | | |
| □ Don't know/Refuse to answer ••••🡺 **I23** | | | | |
| **I22c Record answer** ______ | | | | |
| □ Don't know/Refuse to answer ••••🡺 **I23** | | | | |
| **I22d Record answer** ______ | | | | |
| □ Don't know/Refuse to answer ••••🡺 **I23** | | | | |
| **I22e Record answer** ______ | | | | |
| □ Don't know/Refuse to answer | | | | |
| **I23 Now please tell me, what were the three things I asked you to remember earlier?** | | | | |
| □ Ball □ Flag □ Tree  □ None of the above □ Not assessed | | | | |
| **I24 What is this?** | | | | |
| □ Correct □ Incorrect □ Not Assessed | | | | |
| **I25 What is this?** | | | | |
| □ Correct □ Incorrect □ Not Assessed | | | | |
| **I26 Now please repeat the sentence I say. The sentence is “Forty-four stone lions.”** | | | | |
| □ Correct □ Incorrect □ Not Assessed | | | | |
| **I27** **Please follow the instructions on this card.** | | | | |
| □ Respondent closed eyes; Correct | | | | |
| □ Interviewer read aloud the sentence on the card, then respondent closed eyes: Correct | | | | |
| □ Error/Omission (e.g., respondent did not close eyes) | | | | |
| □ Not assessed | | | | |
| **I28 I will now give you a piece of paper. Please pick up the paper with your right hand, fold it in half with both hands, and place the folded paper on your left leg.** | | | | |
| **I28a** **Hand** | | | | |
| □ Respondent picked up the paper with their right hand, correct | | | | |
| □ Respondent did not pick up the paper with their right hand, incorrect | | | | |
| □ Not assessed | | | | |
| **I28b Folding Paper** | | | | |
| □ Respondent folded the paper with both hands, correct | | | | |
| □ Respondent did not fold paper with both hands, incorrect | | | | |
| □ Not assessed | | | | |
| **I28c** **Leg** | | | | |
| □ Respondent placed folded paper on left leg, correct | | | | |
| □ Respondent did not place folded paper on left leg, incorrect | | | | |
| □ Not assessed | | | | |
| **I29 Please write a complete, meaningful sentence.** | | | | |
| □ Correct □ Incorrect □ Not Assessed | | | | |
| **I30 Here is a picture. Please draw it here exactly as shown.**  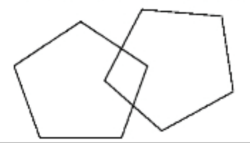 | | | | |
| □ Correct □ Incorrect □ Not Assessed | | | | |
| **5. Fall-Related** | | | | |
| **I31 Have you had any falls or slips in the past month?** | | | | |
| □ Yes □ No | | | | |
| **I32 Have you had any falls in the past year? This includes any falls or slips.** | | | | |
| □ Yes | | | | |
| □ No ••••🡺 **Outcome Section** | | | | |
| **I33 Location of fall (multiple selections possible):** | | | | |
| □Home a. Living room ••••🡺 **I34A**  b. Bedroom ••••🡺 **I34A**  c. Kitchen ••••🡺 **I34A**  d. Bathroom ••••🡺 **I34A**  e. Balcony ••••🡺 **I34A**  f. Stairs ••••🡺 **I34B**  g. Other ••••🡺 **I34A** | | | | |
| □ Stairs in the neighborhood or outdoors ••••🡺 **I34B** | | | | |
| □ Public residential areas (excluding stairs) ••••🡺 **I34C**  □ Sports and athletic facilities (excluding stairs) ••••🡺 **I34C** | | | | |
| □ Commercial and service facilities (excluding stairs) ••••🡺 **I34C**  □ Roads/streets (excluding stairs) ••••🡺 **I34C** | | | | |
| □ Farmland ••••🡺 **I34C**  □ Forests ••••🡺 **I34C** | | | | |
| □ Other **••••🡺 I34A / I34B /I34C /I34D**  □ Unknown **••••🡺 I34D** | | | | |
| **I34 Risk factors in the home environment related to this injury (multiple selections possible):** | | | | |
| **I34A Causes of indoor falls:** ••••🡺 **I34D** | | | | |
| □ Flooring material is not slip-resistant, with protrusions, depressions, or uneven surfaces. | | | | |
| □ Obstacles such as clutter or electrical cords on the floor causing accidental falls. | | | | |
| □ Insufficient lighting in the home, leading to accidental falls. | | | | |
| □ Narrow passageways in areas like the bathroom, bedroom, dining room, or kitchen, leading to accidental falls. | | | | |
| □ Carpets or rugs in the home, leading to accidental falls. | | | | |
| □ Water on the floor in the home, leading to accidental falls. | | | | |
| □ Lack of non-slip mats or accessible grab bars in the bathroom, leading to accidental falls. | | | | |
| □ Kitchen lacks non-slip mats and accessible handrails, causing accidental falls. | | | | |
| □ Furniture is unstable or unsecured, leading to tipping or overturning and falls. | | | | |
| **I34B** **Stair Fall Causes:** ••••🡺 **I34D** | | | | |
| □ Stair handrails are unstable, improperly sized, or lack non-slip grips. | | | | |
| □ Stair treads uneven, non-slip surfaces lacking, edges without clear marking. | | | | |
| □ Stairway entrance without guardrails or safety gates, causing accidental falls. | | | | |
| **I34C Community or Outdoor Fall Causes:：** ••••🡺 **I34D** | | | | |
| □ Rough, uneven, or slippery walkway surfaces prone to slips and falls. | | | | |
| □ Presence of artificial lakes, ponds, or other bodies of water with swift currents and varying depths. | | | | |
| □ Inadequate lighting in the community environment leading to falls or stumbles. | | | | |
| □ Poorly planned vehicle lanes and parking spaces in the community environment creating accident hotspots. | | | | |
| □ Overcrowding of pedestrians in the community causing falls or stumbles. | | | | |
| □ Steps on walkways cause falls. | | | | |
| **I34D** **Lack of assistive devices, wearing/using devices:** | | | | |
| □ Non-slip shoes not worn, leading to accidental falls. | | | | |
| □ Going out without assistive devices like canes or walkers, causing accidental falls. | | | | |
| □ Lack of family accompaniment  □Lack of hearing aids, eyeglasses | | | | |
| **I35 Activity at time of injury (multiple selections possible):** | | | | |
| □ Work □ Housework □ Study □ Sports activities  □ Leisure activities (e.g., going out to play) □ Driving or riding in a vehicle  □ Other □ Unknown | | | | |
| **I36 Injured body part (multiple selections possible):** | | | | |
| □ Head □ Neck □ Chest □ Shoulder  □ Back □ Waist □ Hip □ Abdomen  □ Limbs [a Knee joint b Ankle joint c Foot d Upper limb muscles e Lower limb muscles f Wrist joint g Elbow joint]  □ Multiple areas throughout body, difficult to describe | | | | |
| **I37 Injury Type (multiple selections possible):** | | | | |
| □ Fracture □ Superficial wound □ Ligament sprain □ Ligament tear  □ Muscle strain □ Muscle tear □ Internal organ injury □ Open wound | | | | |
| □ Other | | | | |

| **Part X Oral Health Status and Behavior** |
| --- |
| 1. **Basic Information** |
| **J1 How would you rate your dental and oral health?**  □ Excellent  □ Good  □ Fair  □ Poor  □ Very poor |
| **2. Sugar Intake** |
| **J2 How often do you snack between meals each day?**  □ Never  □ 1-2 times  □ 3-4 times  □ 5-6 times  □ More than 6 times  □ More than 6 time |
| **J3 How often do you typically consume the following foods or beverages?**   \|  \| Daily≥2times \| Daily 1times \| Weekly 2-6times \| Weekly 1times \| Monthly 1-3times \| Rarely/Never \| \| --- \| --- \| --- \| --- \| --- \| --- \| --- \| \| J3a Sweet snacks (cookies, cakes, pastries) and candies (chocolate, sugary gum) \| □ \| □ \| □ \| □ \| □ \| □ \| \| J3b Sweetened beverages (sweetened drinks, carbonated drinks like cola, orange juice, apple juice, lemonade, etc., non-freshly squeezed juices) \| □ \| □ \| □ \| □ \| □ \| □ \| \| J3c Sweetened milk, yogurt, powdered milk, tea, soy milk, coffee \| □ \| □ \| □ \| □ \| □ \| □ \| |
| **3. Oral Health Practices** |
| **J4 Which of the following oral health issues do you currently have? (Select all that apply)**  □ Cavities/Tooth decay  □ Tartar/Dental plaque buildup  □ Bleeding gums  □ Mouth ulcers  □ Missing teeth  □ Broken teeth  □ Dry mouth  □ Other: _________ (Please specify)  □ Don't know  □ No oral health issues |
| **J5 How often do you brush your teeth?**  □ ≥2 times daily  □ Once daily  □ 2-6 times weekly  □ Once weekly  □ 1-3 times per month  □ Rarely/Never |
| **J5a If you brush your teeth regularly, what method do you typically use?**  □ No specific method  □ Horizontal brushing method  □ Vertical brushing method  □ Rotating brushing method  □ Mixed brushing method |
| **J6 Do you wear removable dentures (e.g., full dentures, partial dentures)?** |
| □ Yes ••••🡺 **J6a**  □ No ••••🡺 **J7** |
| **J6a If yes, how often do you clean your dentures?**  □ Never  □ Occasionally  □ Frequently  □ Once/day  □ 2-3 times/day |
| **J7 Did you visit the dentist in the past year?** |
| □ Yes ••••🡺 **J7a**  □ No. ••••🡺 **J8** |
| **J7a Total cost of dental visits in the past year: __________ yuan? (Enter a whole number. Write “N” if unknown or refusing to answer)** |
| **J7b Your personal payment share of the above dental costs: ________ % (Enter a whole number. Write “N” if unknown or refusing to answer)** |
| **J7c Reimbursement method for last dental visit? (Multiple selections allowed)**  □ Urban Employee Basic Insurance □ Urban Resident Basic Medical Insurance  □ New Rural Cooperative Medical Scheme □ Commercial Insurance  □ Public Medical Expense Reimbursement □ No reimbursement (fully self-paid)  □ Other reimbursement method: _________(Please specify) |
| 1. **GOHAI** **Geriatric Oral Health Assessment Index (GOHAI)** |
| **J8 How much do oral problems affect the following aspects of your life?**   \|  \| Very often \| Often \| Sometimes \| Rarely \| Not at all \| \| --- \| --- \| --- \| --- \| --- \| --- \| \| J8a Do you often limit the types or amounts of food you eat because of your teeth or dentures? \| □ \| □ \| □ \| □ \| □ \| \| J8b Do you have difficulty biting or chewing food? \| □ \| □ \| □ \| □ \| □ \| \| J8c Do you often feel discomfort or difficulty swallowing food? \| □ \| □ \| □ \| □ \| □ \| \| J8d Do your teeth or dentures interfere with your speech? \| □ \| □ \| □ \| □ \| □ \| \| J8e Do you often feel discomfort in your mouth while eating? \| □ \| □ \| □ \| □ \| □ \| \| J8f Do you often limit your interactions with others because of your teeth or dentures? \| □ \| □ \| □ \| □ \| □ \| \| J8g Are you often dissatisfied or unhappy with the appearance of your teeth, gums, or dentures? \| □ \| □ \| □ \| □ \| □ \| \| J8h Do you often use medication to relieve oral pain or discomfort? \| □ \| □ \| □ \| □ \| □ \| \| J8i Do you often worry about or focus on problems with your teeth, gums, or dentures? \| □ \| □ \| □ \| □ \| □ \| \| J8j Do you often feel nervous or unnatural around others due to problems with yo \| □ \| □ \| □ \| □ \| □ \| \| J8k Do you often feel uncomfortable eating in front of others due to problems with your teeth or dentures? \| □ \| □ \| □ \| □ \| □ \| \| J8l Are your teeth or gums sensitive to cold, hot, or sweet stimuli? \| □ \| □ \| □ \| □ \| □ \| |
| **5.** **Family Situation** |
| **J9 Do you currently live with your children?**  □ Yes, living with children  □ No, living alone  □ No, living with spouse/friends  □ No, living in nursing home/assisted living |
| **J9a How concerned are your children about your oral health?**  □ Not concerned  □ Somewhat concerned  □ Concerned  □ Fairly concerned  □ Very concerned |
| **6.** **Chewing Ability** |
| **J10 Which of the following best describes your experience chewing these foods?**   \|  \| Very easy to chew \| Difficult to chew \| Unable to chew \| \| --- \| --- \| --- \| --- \| \| J10a Hard foods like dried squid/shredded squid/beef jerky \| □ \| □ \| □ \| \| J10b Whole apple \| □ \| □ \| □ \| \| J10c Hard, crunchy peanuts (e.g., raw/roasted peanuts) \| □ \| □ \| □ \| \| J10d Hard pickled radish/dried vegetables \| □ \| □ \| □ \| \| J10e Hard biscuits \| □ \| □ \| □ \| \| J10f Vegetable stalks \| □ \| □ \| □ \| \| J10g Soft biscuits/dumplings, etc. \| □ \| □ \| □ \| \| J10h Ham sausages/fish balls/konjac, etc. \| □ \| □ \| □ \| \| J10i Noodles \| □ \| □ \| □ \| \| J10j Rice \| □ \| □ \| □ \| |

**Part XI: The Outcome Section**

**Y1 How long ago was your most recent lipid profile test?**

□ Within 6 months □ Within 12 months □ Within 2 years

□ More than 2 years ago □ Never had a lipid profile test □ Can't recall

**Y2 How long ago was your most recent pulmonary function test?**

□ Within 6 months □ Within 12 months □ Within 2 years

□ More than 2 years ago □ Never had a lipid profile test □ Can't recall

| **Y3** **Since your last baseline survey, have you been diagnosed with any disease by a doctor at a township/district-level hospital or higher?** |
| --- |
| □ Yes ••••🡺 **Y3.1**  □ No ••••🡺 **Y4** |

**Y3.1 What new disease? (Multiple selections)**

□ Hypertension

Date of initial diagnosis: Year Month Hospital where first diagnosed:

Hospitalization: □ Yes □ No

Antihypertensive medication use: □ Regularly □ Intermittently □ Never

Current blood glucose levels: □ Normal □ Not normal □ Unknown

□ Diabetes

Date of initial diagnosis: Year Month Hospital where first diagnosed:

Hospitalization: □ Yes □ No

Hypoglycemic medication use: □ Regularly □ Intermittently □ Never

Current blood glucose levels: □ Normal □ Not normal □ Unknown

□ Hyperlipidemia

Date of initial diagnosis: Year Month Hospital where first diagnosed:

Hospitalization: □ Yes □ No

□ Coronary Heart Disease

Date of initial diagnosis: Year Month Hospital where first diagnosed:

Hospitalization: □ Yes □ No

□ Stroke

Date of initial diagnosis: Year Month Hospital where first diagnosed:

Hospitalization: □ Yes □ No

□ Chronic Obstructive Pulmonary Disease (COPD)

Date of initial diagnosis: Year Month Hospital where first diagnosed:

Hospitalization: □ Yes □ No

□ Rheumatic Heart Disease

Date of initial diagnosis: Year Month Hospital where first diagnosed:

Hospitalization: □ Yes □ No

□ Pulmonary Heart Disease

Date of initial diagnosis: Year Month Hospital where first diagnosed:

Hospitalization: □ Yes □ No

□ Pulmonary Tuberculosis

Date of initial diagnosis: Year Month Hospital where first diagnosed:

Hospitalization: □ Yes □ No

□ Chronic Bronchitis/Emphysema

Date of initial diagnosis: Year Month Hospital where first diagnosed:

Hospitalization: □ Yes □ No

□ Asthma

Date of initial diagnosis: Year Month Hospital where first diagnosed:

Hospitalization: □ Yes □ No

□ Chronic Hepatitis/Cirrhosis

Date of initial diagnosis: Year Month Hospital where first diagnosed:

Hospitalization: □ Yes □ No

□ Gastrointestinal Ulcer

Date of initial diagnosis: Year Month Hospital where first diagnosed:

Hospitalization: □ Yes □ No

□ Chronic Gastroenteritis

Date of initial diagnosis: Year Month Hospital where first diagnosed:

Hospitalization: □ Yes □ No

□ Gallstones

Date of initial diagnosis: Year Month Hospital where first diagnosed:

Hospitalization: □ Yes □ No

□ Cholecystitis

Date of initial diagnosis: Year Month Hospital where first diagnosed:

Hospitalization: □ Yes □ No

□ Fracture

Date of initial diagnosis: Year Month Hospital where first diagnosed:

Hospitalization: □ Yes □ No

□ Rheumatoid Arthritis

Date of initial diagnosis: Year Month Hospital where first diagnosed:

Hospitalization: □ Yes □ No

□ Gout

Date of initial diagnosis: Year Month Hospital where first diagnosed:

Hospitalization: □ Yes □ No

□ Intervertebral Disc Disease

Date of initial diagnosis: Year Month Hospital where first diagnosed:

Hospitalization: □ Yes □ No

□ Psychiatric/Psychological Disorders

Date of initial diagnosis: Year Month Hospital where first diagnosed:

Hospitalization: □ Yes □ No

□ Neurasthenia

Date of initial diagnosis: Year Month Hospital where first diagnosed:

Hospitalization: □ Yes □ No

□ Traumatic Brain Injury

Date of initial diagnosis: Year Month Hospital where first diagnosed:

Hospitalization: □ Yes □ No

□ Hyperthyroidism

Date of initial diagnosis: Year Month Hospital where first diagnosed:

Hospitalization: □ Yes □ No

□ Hypothyroidism

Date of initial diagnosis: Year Month Hospital where first diagnosed:

Hospitalization: □ Yes □ No

□ Thyroid Nodules

Date of initial diagnosis: Year Month Hospital where first diagnosed:

Hospitalization: □ Yes □ No

□ High-Altitude Polycythemia

Date of initial diagnosis: Year Month Hospital where first diagnosed:

Hospitalization: □ Yes □ No

□ High-Altitude Cardiomyopathy

Date of initial diagnosis: Year Month Hospital where first diagnosed:

Hospitalization: □ Yes □ No

□ Mixed Chronic High Altitude Disease

Date of initial diagnosis: Year Month Hospital where first diagnosed:

Hospitalization: □ Yes □ No

□ Sleep Apnea-Hypopnea Syndrome

Date of initial diagnosis: Year Month Hospital where first diagnosed:

Hospitalization: □ Yes □ No

□ Malignant Tumor*

Date of initial diagnosis: Year Month Hospital where first diagnosed:

Hospitalization: □ Yes □ No

□ Chronic Kidney Disease

Date of initial diagnosis: Year Month Hospital where first diagnosed:

Hospitalization: □ Yes □ No

□ Heart Failure

Date of initial diagnosis: Year Month Hospital where first diagnosed:

Hospitalization: □ Yes □ No

*Specify specific location □ (1 to 12)

*If malignant tumor, indicate specific location number in the box: (If more than one location, list the primary tumor site)

1. Lungs, 2. Esophagus, 3. Stomach, 4. Liver, 5. Intestines, 6. Breast, 7. Prostate, 8. Cervix, 9. Uterus 10. Ovaries 11. Thyroid, 12. Other

**Y4. Have you used antibiotics within the past 30 days?**

□ Yes □ No
