# Supplementary material for: Machine learning model integrating oral microbiota and clinical features for predicting osteoporosis and bone loss in high-altitude populations
Source: BMC Microbiol. 2026 Mar 5;26:337. doi: 10.1186/s12866-026-04718-0 (PMC13072478; doi:10.1186/s12866-026-04718-0)
Supplement: Supplementary file 2 — Additional file 2: Table S1. Performance comparison of the primary and sensitivity models on training and test sets. FigS1. Alpha rarefaction curves confirming sequencing depth sufficiency. FigS2. Zero-inflation diagnostics and prevalence filtering of microbial features. Fig. S3. Variance Inflation Factors (VIF) confirming absence of multicollinearity. Fig. S4. Correlation matrix of continuous predictors selected by elastic-net regression. Fig. S5. Confusion matrices of five machine-learning models on the test set. Fig. S6. Ten-fold cross-validation performance of the logistic-regression model. Fig. S7. Exploratory mediation analysis illustrating the indirect effect of Abiotrophia. Fig. S8. Sensitivity analysis curves demonstrating stable and monotonic predictor effects. [file 12866_2026_4718_MOESM2_ESM.docx]

Supplementary Results

**Supplementary Information Summary**
Table.S1. Performance comparison of the primary and sensitivity models on training and test sets.
Fig. S1. Alpha rarefaction curves confirming sequencing depth sufficiency.
Fig. S2. Zero-inflation diagnostics and prevalence filtering of microbial features.
Fig. S3. Variance Inflation Factors (VIF) confirming absence of multicollinearity.

Fig. S4. Correlation matrix of continuous predictors selected by elastic-net regression.
Fig. S5. Confusion matrices of five machine-learning models on the test set.
Fig. S6. Ten-fold cross-validation performance of the logistic-regression model.
Fig. S7. Exploratory mediation analysis illustrating the indirect effect of *Abiotrophia*.
Fig. S8. Sensitivity analysis curves demonstrating stable and monotonic predictor effects.

**Table.S1. Performance comparison of the primary and sensitivity models on training and test sets.**

| Model | Dataset | Accuracy | Sensitivity | Specificity | Precision | F1 | AUC |
| --- | --- | --- | --- | --- | --- | --- | --- |
| Main Model (NR vs OP&BL) | Train | 0.819 | 0.837 | 0.802 | 0.809 | 0.822 | 0.886 (0.857–0.914) |
| Sensitivity Model (NR vs BL) | Train | 0.833 | 0.852 | 0.814 | 0.821 | 0.836 | 0.899 (0.871–0.922) |
| Main Model (NR vs OP&BL) | Test | 0.874 | 0.735 | 0.914 | 0.706 | 0.720 | 0.885 (0.823–0.937) |
| Sensitivity Model (NR vs BL) | Test | 0.846 | 0.725 | 0.874 | 0.569 | 0.637 | 0.865 (0.797–0.927) |

Performance metrics from the sensitivity analysis support the merging strategy. The main model achieved an AUC value of 0.885 (0.823–0.937) on the test set, while the sensitivity model (NR vs BL) demonstrated comparable performance (AUC = 0.865, 0.797–0.927), indicating consistent performance even when focused on the boneloss group. These findings suggest that the selected predictors maintain discriminative ability across the spectrum of bone health impairment, supporting the model's clinical applicability in identifying individuals at risk for both early and late-stage bone deterioration.


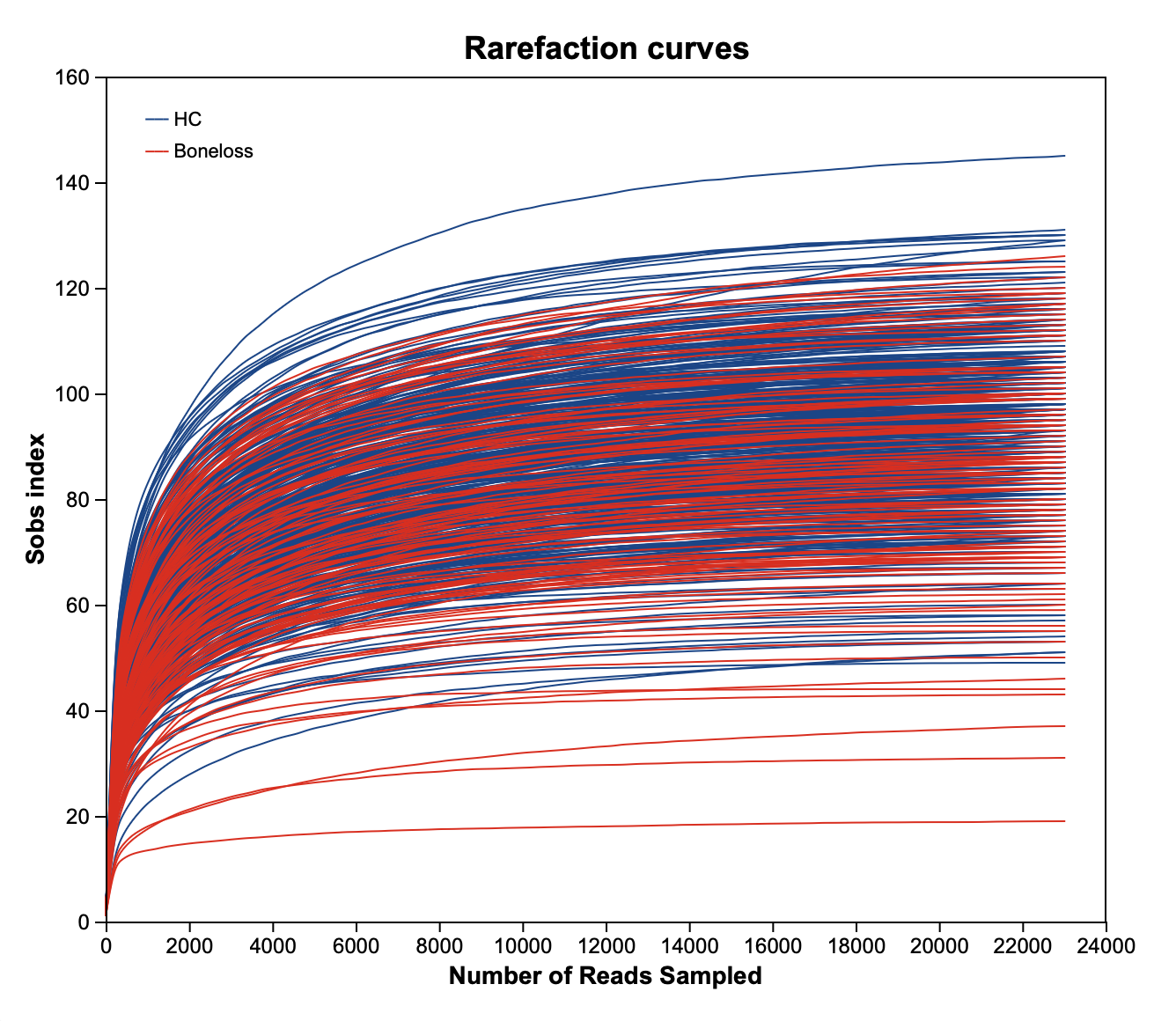


**Fig.S1. Rarefaction curve.** Dilution curves normalized to 23,033 sequences per sample for a total of 12,898,480 reads.

**Fig.S2. Zero inflation diagnostic for microbiome features.** (A) Histogram showing the distribution of zero proportions across all genera present in the rarefied abundance table. The red dashed line indicates the prevalence filter threshold (≥10% sample presence). Genera to the left of the line were retained for downstream analysis. (B) Box plot comparing zero proportions between the retained and excluded genera.

After rarefaction, 474 genera had non-zero abundance in at least one sample. Applying the prevalence filter (≥10%) retained 148 genera for downstream analysis (e.g., MaAsLin2 and predictive modeling), while excluding 326 low-prevalence genera. The retained genera had a median zero proportion of 42% (IQR: 28–59%), indicating that extreme zero inflation was controlled in the final analytical dataset

**Fig.S3. Variance Inflation Factor (VIF) of variables after collinearity screening**. Categorical variables were processed as follows: dichotomous variables (e.g., Gender) were retained as 0/1; nominal multicategorical variables (e.g., A5 / Current marital status) were dummy-coded using a one-hot scheme (baseline level omitted to avoid the dummy variable trap). Multicollinearity was diagnosed in two steps: First, continuous and binary variables with VIF > 5 were excluded. Second, for each set of dummy variables, if any variable within the set exhibited VIF > 5, only the category explaining the greatest variance was retained; the others were excluded to ensure final VIF values for all features remained below 3. This figure presents the VIF values for all final variables entering the model, confirming successful control of multicollinearity.


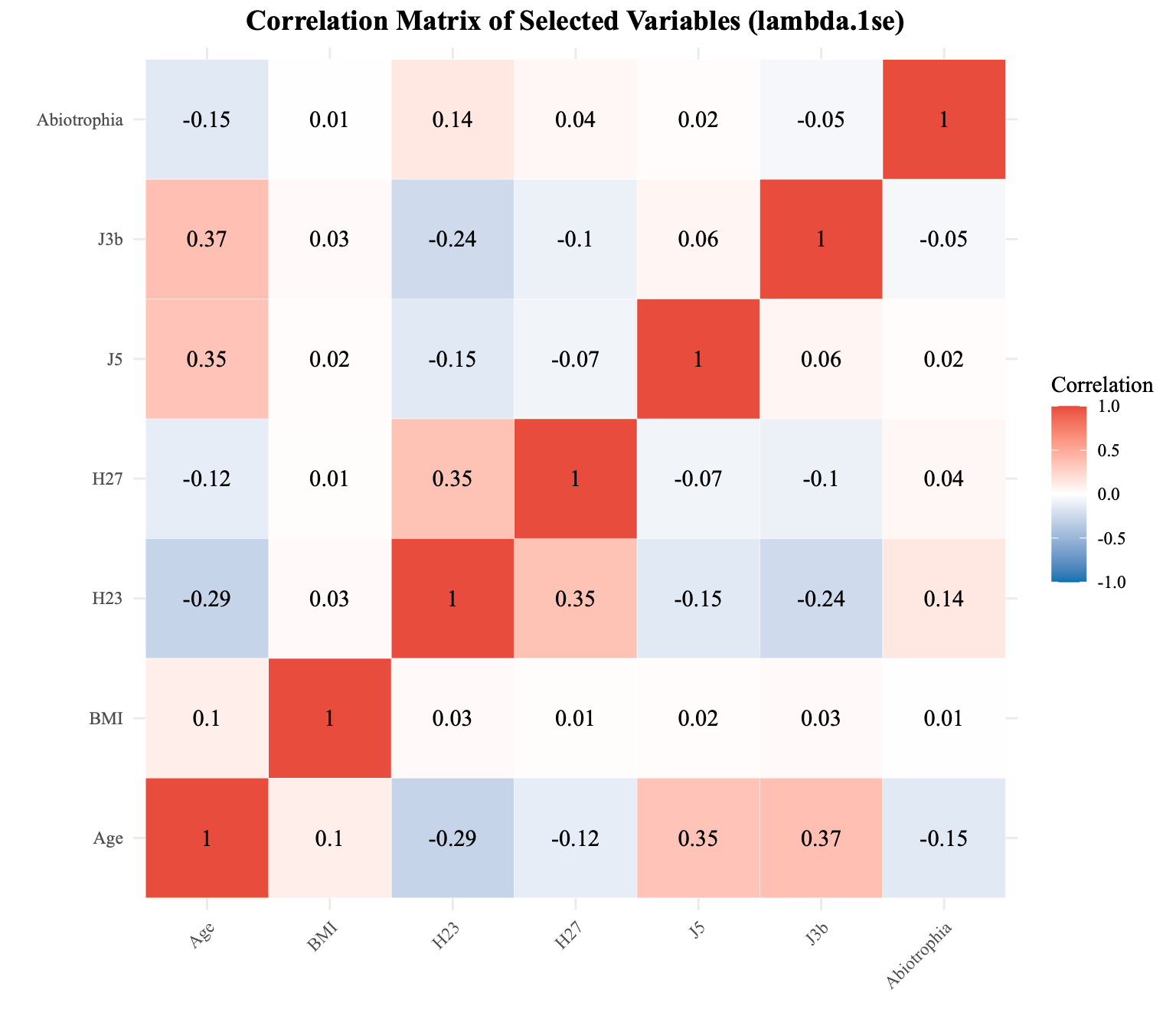


**Fig.S4. Correlation matrix of predictors selected by elastic net regression (λ.1se).**

This matrix displays the pairwise Spearman correlation coefficients (r) among the seven continuous predictors retained after elastic net regularization. Color intensity and numerical values indicate the strength and direction of each correlation. The strongest correlation was observed between age and Frequency of sweet-drink consumption (J3b)(|r| = 0.37); all other correlations were weak (|r| < 0.35). Together, these results confirm the absence of severe multicollinearity among the selected features, supporting the stability and validity of the final model.

**Fig.S5. Confusion matrices for the five machine learning models in the test set.** (A) Logistic regression (LR); (B) Naive Bayes (NB); (C) Random forest (RF); (D) Support vector machine (SVM); (E) Extreme Gradient Boosting (XGB). Among the five candidate models, LR achieved the best balance with 73.5 % sensitivity and 91.4 % specificity on

the test set, and was therefore selected as the final model.

**Fig.S6.** **Results of 10-fold cross-validation of the logistic regression model.** The LR model demonstrated robust stability across folds: the mean AUC was 0.849 ± 0.082, and the Kappa statistic reached 0.443, indicating moderate agreement with reference diagnoses under Landis & Koch criteria. Notably, 90% of folds exceeded the clinically acceptable AUC threshold of 0.75, and 70% surpassed 0.820, reflecting consistently strong discriminative ability.


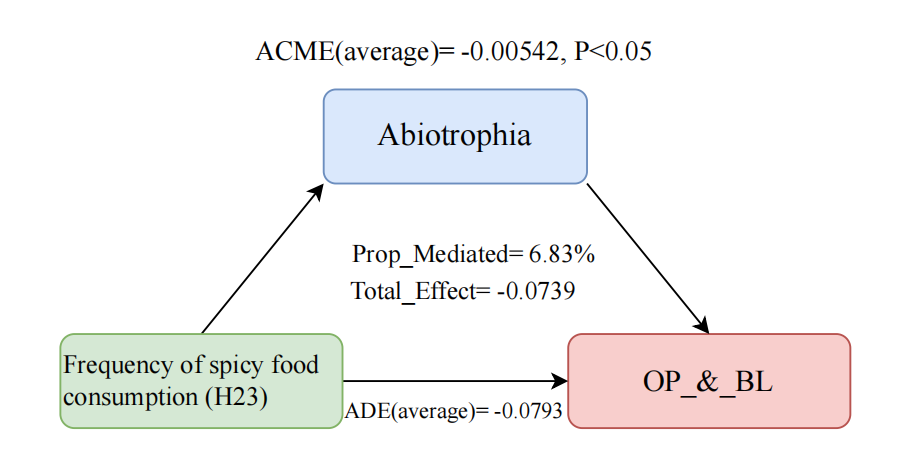


Fig.S7. The mediation effect of *Abiotrophia* in the relationship between Frequency of spicy food consumption (H23) and osteoporosis and bone loss (OP&BL). ACME, The average causal mediation effect; ADE, The average direct effect. Proportion of mediation = ACME (average)/Total effect. ACME represents the effect of frequency of spicy food consumption (H23) on OP&BL through mediator. ADE refers to the direct effect of frequency of spicy food consumption (H23) on OP&BL.

**Fig.S8.** **Sensitivity analysis of logistic regression predictions to ±2 SD perturbations in input variables**. (A) Combined effect when all variables vary simultaneously; (B)–(H) Univariate sensitivity for Age, BMI, *Abiotrophia*, frequency of spicy food consumption (H23), Tooth brushing frequency (J5), Frequency of sweet-drink consumption (J3b), Current marital status (Separated/Divorced, A5_3) and frequency of numbing food consumption (H27).Sensitivity analysis confirmed model robustness and clinical interpretability: Each continuous predictor was perturbed by ±2 standard deviations (SD) across the test set while holding all other variables constant. Age exerted the strongest influence: a +2 SD increase raised the predicted probability from 0.067 to 0.854 (Δ = +0.787), whereas BMI generated the largest negative shift (Δ = –0.223). *Abiotrophia*, Tooth brushing frequency (J5), and frequency of spicy food consumption (H23) produced weaker inverse associations (Δ = –0.147, –0.117 and –0.098, respectively), while Frequency of sweet-drink consumption (J3b) and frequency of numbing food consumption (H27) conferred minimal positive changes (Δ = +0.029 and +0.006). All response curves remained monotonic and non-overlapping, confirming model robustness and clinical interpretability.
